# The ethnomedicine, phytochemistry, and pharmacological properties of the genus Bersama: current review and future perspectives

**DOI:** 10.3389/fphar.2024.1366427

**Published:** 2024-03-21

**Authors:** Gashaw Nigussie, Sintayehu Ashenef, Asfaw Meresa

**Affiliations:** Traditional and Modern Medicine Research and Development Directorate, Armauer Hansen Research Institute, Addis Ababa, Ethiopia

**Keywords:** *Bersama* genus, antioxidant, antimalarial, antidiabetic, antiviral, cytotoxic, mangiferin, quercetin-3-O-araiopyraoside

## Abstract

Bersama (Melianthaceae) has been used in traditional medicine for a wide range of ailments, including blood purifier, immune booster, psychotropic medication, and treatment for malaria, hepatitis, infertility, diabetes, impotency, meningitis, and stroke. This review gathers fragmented information from the literature on ethnomedicinal applications, phytochemistry, pharmacology, and toxicology of the Bersama genus. It also explores the therapeutic potential of the Bersama genus in ethnophytopharmacology, allowing for further investigation. All the available information published in the English language on Bersama genus was compiled from electronic databases such as Academic Journals, Ethnobotany, Google Scholar, PubMed, Science Direct, Web of Science, and library search using the following keywords: “Bersama genus,” “traditional use,” “phytochemistry,” “pharmacological effects,” and “toxicology”. The ethnomedical applications of the Bersama genus have been recorded, and it has been used traditionally for more than 30 different types of ailments. Thus far, more than 50 compounds have been isolated from the genus. Cardiac glycosides and terpenoids are the main compounds isolated from the *Bersama* genus. Different plant parts of *Bersama* genus extracts demonstrated a wide range of pharmacological properties, including antioxidant, antimalarial, antidiabetic, antiviral, anti-inflammatory, and cytotoxic activity. Exemplary drug leads from the genus include mangiferin and quercetin-3-*O*-arabinopyranoside, both of which have antioxidant activities. *Bersama* genus has long been used to cure a wide range of ailments. *Bersama* genus extracts and phytochemicals have been found to have promising pharmacological activities. Further study on promising crude extracts and compounds is required to develop innovative therapeutic candidates.

## 1 Introduction

Medical plants are essential parts of medicine and have a significant role in local healthcare systems, especially for rural people (Tola et al., 2023). Ethnic cultures are closely associated with plant knowledge and use. Geographical location, climatic conditions, and quantity of medicinal plants all influence their distribution, taxonomic variety, and abundance, and different communities have different ethnomedicinal treatment systems (Farooq et al., 2019). Approximately more than 80% of the world’s population employs traditional and complementary medicine (WHO, 2013). The report shows that medicinal plants are being researched as a complementary therapy and as a support for medical treatments. Before the advent of modern medicine, indigenous knowledge that was passed down through generations contained health-related elements that are now included in traditional medicine. Traditional medicine is described by the WHO as an array of abilities, knowledge, and practices based on theories, beliefs, and indigenous wisdom of various cultures for the prevention, diagnosis, improvement, and treatment of mental and physical disorders (WHO, 2013). Traditional medicine, which primarily uses plants, has frequently been supported by phytochemical investigations, pharmacological studies, and clinical trials, sparking further study on medicinal plants in various parts of the world (Nigussie et al., 2022). To assure the effectiveness and safety of traditional medicine and the practices used by practitioners and patients of traditional medicine, a more in-depth investigation is required. Traditional medications, on the other hand, have the potential to have negative side effects. In order to support member nations in strengthening the role of traditional medicine in maintaining public health, WHO developed strategy plan (WHO, 2013). Most member nations are currently implementing this strategic plan (WHO, 2013).

**FIGURE 1:**
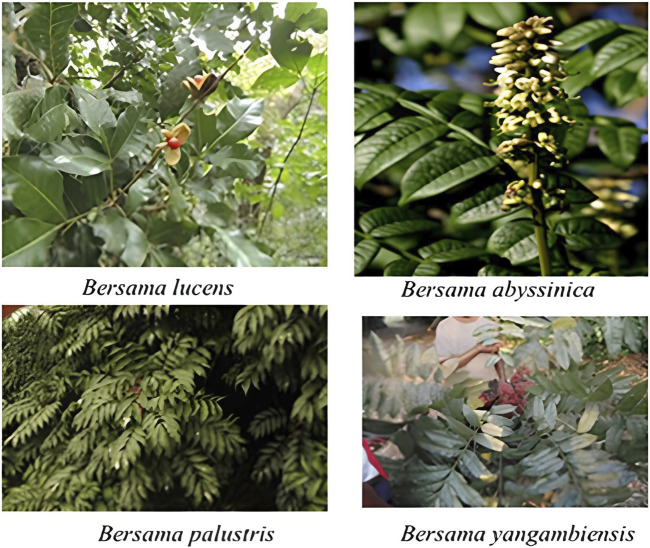
Images of some representative samples of *Bersama* genus.

*Bersama* is a genus of eight species that are found throughout tropical and subtropical Africa (Acharyulu et al., 2014). It consists of trees and shrubs from the Melianthaceae family. *Bersama abyssinica* Fresen., *Bersama lucens* (Hochst.) Szyszył., *Bersama palustris* L. Touss., *Bersama stayneri* Phillips, *Bersama swinnyi* Phillips, *Bersama swynnertonii* Baker f., *Bersama tysoniana* Oliv., and *Bersama yangambiensis* L. Touss. are among the recognized species. *Bersama* is the Ethiopian name for this genus (Verdcourt, 1957; Baijnath, 2021). This genus includes evergreen shrubs to small trees up to 12 m tall with grey or brown bark that are found in the Democratic Republic of the Congo, Tanzania, Mozambique, Zambia, Zimbabwe, Angola, Nigeria, Ethiopia, Kenya, Sudan, and Uganda (Djemgou et al., 2010). It grows from sea level up to 2,700 m altitude in lowland bush savanna and gallery forests. The genus is employed by local communities to treat microbiological illnesses, especially mycobacterial infections (Geyid et al., 2005). *Bersama* genus have been used extensively and routinely by society and traditional healers for a variety of ailments. The leaves, stems, and roots are utilized in the preparation of traditional medicine. Bersama genus exhibited an extensive range of biological activities and chemical compounds (Amit et al., 2010). According to the World Health Organization’s traditional medicine report, traditional medicine and complementary products, practices, and practitioners will continue to be in high demand (WHO, 2013). However, there is a lack of knowledge documentation. Since no comprehensive review study on the diverse aspects of the genus *Bersama* has been published to date, this review focuses on ethnomedicinal use, phytochemistry, and pharmacological activities. The representative samples of *Bersama* genus are presented in Figure 1 (Mbuvi et al., 2019; Agidew, 2022).

## 2 Review methodology

For this review, a comprehensive literature search was conducted up to 28 February 2023, traditional use, phytochemical constituents, pharmacology, toxicological studies of *Bersama* genus. GN and AM retrieved all of the information that was available on *Bersama* genus from library documents and online databases (PubMed, Web of Science, Wiley, Science Direct, Elsevier, Scopus, Frontiers, ACS publications, SciFinder, and Google Scholar). The keywords used to search were: “*Bersama* genus,” “traditional use,” “phytochemistry,” “pharmacological effects,” and “toxicology”. Most of the cited information in this review were taken from peer-reviewed journals and were published in English. Information of related books, PhD and MSc dissertations were also used as references. After retrieving articles, books, and Ph.D. and MSc dissertations, AM and SA carefully collected data about the pharmacological properties, phytochemistry, and ethnomedical uses of *Bersama* genus. Reviews with unclear validity, papers in languages other than English, papers that did not have significant findings were excluded, while studies with high relevance were critically appraised. The worldwide plant name index (https://www.ipni.org) and the Kew Botanical Garden plant name database (https://www.kew.org) were used to validate species names and their synonyms are presented in Table 1.

**TABLE 1:** Synonyms of *Bersama* genus.

Number	Species	Synonyms
1	*Bersama abyssinica* Fresen.	*Bersama schreberifolia*
		*Bersama schweinfurthii*
		*Bersama serrata*
		*Bersama volkensii*
2	*Bersama lucens* (Hochst.) Szyszył.	Homotypic Synonyms (*Natalia lucens* Hochst.)
		Heterotypic Synonyms (*Rhaganus lucidus* E.Mey.)
3	*Bersama palustris* L.Touss.	-
4	*Bersama stayneri* Phillips	*Bersama tysoniana*
5	*Bersama swinnyi* Phillips	-
6	*Bersama swynnertonii* Baker f.	-
7	*Bersama tysoniana* Oliv.	Heterotypic Synonyms (*Bersama stayneri* E.Phillips and *Bersama transvaalensis* Turrill
8	*Bersama yangambiensis* L.Touss.	-

## 3 Result and discussion

### 3.1 Ethnomedicinal uses

The genus is well known for its therapeutic properties. Decoctions of *B. lucens* bark have been widely used in traditional South African medicine to treat various ailments, including for mental disorders, purify blood, headache, impotency, leprosy, meningitis, sexually transmitted infections, and tuberculosis (Watt and Breyer-Brandwijk, 1962; Cocks and Dold, 2006; Monakisi, 2007; Bhat, 2013). *B. swinnyi* and *B. tysoniana* also recognized as important sources of traditional treatments for migraines, impotence, infertility, menstrual discomfort, mental disorders, strokes, and venereal illnesses (Hutchings, 1996). These three species are included in the book “medicinal plants of South Africa,” a monographic guide to the most widely used medicinal plants in the nation, including their taxonomy, botanical description, primary medicinal uses, preparation and dosage, active phytochemical compounds, and pharmacological effects (Van Wyk et al., 1997). This inclusion is based on the plants’ popularity as medicines. Additionally, in around two-thirds of South Africa’s provinces—the Eastern Cape, Gauteng, KwaZulu-Natal, Mpumalanga, Northern Cape, and Western Cape provinces—the bark and roots of *B. lucens* are sold as herbal remedies in informal herbal medicine markets (Cocks and Dold, 2006). Traditional medicine has made considerable use of the bark, leaves, and roots of *B. abyssinica* to make decoctions that can be used to treat a variety of issues, including colic, diarrhea, dysentery, and intestinal worms. Additionally, *B. abyssinica* is used to treat rheumatoid arthritis, cancer, diabetes, lumbago, gonorrhea, syphilis, malaria, diabetes, debility, hemorrhoids, and epilepsy (Zekeya et al., 2014). Beer is flavored with powdered *B. abyssinica* stem bark or leaves as an aphrodisiac (Sinan et al., 2021). Leprosy, menstrual cramps, barrenness, and impotence were all treated with a decoction of the leaves and roots (Nakamura et al., 2017). In southern Ethiopia, liver ailments and snake bites were treated using the stem bark of *B. abyssinica* (Kidane et al., 2014). In Kenya, 1 kg of freshly collected *B. abyssinica* bark is boiled in 4 L of water with 5 ripe *Solanum aculeastrum* Dunal seeds, and the resulting infusion is decanted and given to animals suffering from black water fever, east coast fever, and Rift Valley fever daily for 3 days (Amuka et al., 2014). In Uganda, the leaves of *B. abyssinica* are decocted and taken orally to treat diarrhea, stomach discomfort, malaria, syphilis, and gonorrhea (Anywar et al., 2020). The leaves and barks of *B. abyssinca* have been utilized to treat malaria and round warms in Côte d'Ivoire and Cameron by taking the decoction orally (Focho et al., 2009; Guédé et al., 2010). According to reports, *B. abyssinica* plant components are poisonous and have been linked to the deaths of animals (Harker et al., 1962). It is crucial to use the right dosage. The presence of cardiac glycosides, which have an impact on the gastrointestinal, neurological, and respiratory systems, has been linked to *B. abyssinica’s* toxic effects (Ndhlala et al., 2013). For the treatment of diarrhea and roundworm infestations, *B. abyssinica* leaf extracts are taken orally. The plant’s aqueous extract is also used to cure tumors (Asres et al., 2006). The usage of the genus *Bersama* in traditional medicine is summarized in Table 2.

**TABLE 2:** Botanical distribution and ethno-medicinal uses of *Bersema* genus.

Species	Diseases treated	Plant organs used	Preparation and application	Distribution	References
*B. abyssinica*	Malaria	Barks	Decoction of the park part of the plant taken orally	Kenya	Pascaline et al. (2011)
	Microbial infection (black water fever, east coast fever, Rift Valley fever)	Barks	About 1 kg of freshly collected bark is cooked along with 5 ripe seeds of *Solanum aculeastrum* Dunal, in 4 L of water. The resultant infusion is decanted and 1 L given daily for 3 days to animals suffering from black water fever, east coast fever and Rift Valley fever	Kenya	Amuka et al. (2014)
	Rheumatism	Leaves	The fresh leaves crushed, mixed with water and drinking it or wash by it	Ethiopia	Yineger et al. (2008)
	Ascariasis	Leaves	Fresh leaves past taken in empty stomach/fresh leaves boiled with water and drunk for 3 consecutive days	Ethiopia	Birhanu, (2013)
	Tonsillitis	Stem bark	The decoction of stem bark is taken orally	Ethiopia	Giday et al. (2009)
	Malaria	Leaves	The decoction of leave is taken orally	Côte d’Ivoire	Guédé et al. (2010)
	Round worms	Bark	Decoction is taken orally	Cameron	Focho et al. (2009)
	Diabetes	Root bark	Decoction is taken orally	Kenya	Keter and Mutiso, (2012)
	Diarrhoea, stomach ache, malaria, syphilis and gonorrhoea	Leaves	Decoction is taken orally	Uganda	Anywar et al. (2020)
	Wound	Stem	Fresh leafy-stem of this plant is pressed and applied on the affected area	Ethiopia	Siraj et al. (2020)
	Cancer	Bark	The bark is pounded, boiled, and a small amount of the preparation is drunk	Ethiopia	Tuasha et al. (2018)
*Bersama lucens*	Psychoactive	Bark	Tincture of the bark as an emetic	South Africa	Cocks and Dold (2006)
	Blood purifier	Bark	A protective charm; an infusion of bark is taken	South Africa	Cocks and Dold (2006)
	Boost immunity	Stems/leaves	Ground to powder, boiled and drunk	South Africa	Monakisi, (2007)
	Headache	Bark	Bark or root decoctions or tinctures taken orally	South Africa	Pujol (1990), Philander (2011)
	Protective charm (against evil eye, enemies and, lightning)	Bark	-	South Africa	Philander, (2011)
	Hiccough	Bark	Bark infusion taken orally	South Africa	Simon and Lamia, (1991)
	Impotency	Bark	Bark infusion taken orally	Eswatini and South Africa	Watt and Breyer-Brandwijk, (1962)
	Infertility	Bark	Bark infusion taken orally	South Africa	Simon and Lamia (1991), Dweck (2007)
	Leprosy	Bark	Bark infusion applied topically	South Africa	Hutchings (1996), Grace et al. (2003)
	Lice repellent	Stem bark	Stem bark infusion applied topically	South Africa	Buwa and Van Staden (2006), Schemelzer and Gurib-Fakim (2008)
	Meningitis	Stems/leaves	Ground to powder, boiled and drunk	South Africa	Monakisi, (2007)
	Menstrual pain	Bark	Bark or root decoctions or tinctures taken orally	South Africa	Hutchings and van Staden (1994), Van Wyk et al. (1997)
	Stomach problems	Bark	Bark decoctions taken orally	South Africa	Bhat (2013); Khumalo (2018)
	Stroke	Bark/root	Bark or root decoctions or tinctures taken orally	South Africa	Van Wyk et al. (1997), Philander (2011)
	Tuberculosis	Stems/leaves	Ground to powder and boiled, drunk to cure tuberculosis	South Africa	Monakisi, (2007)
	Sexually transmitted infections	Stems/leaves	Ground to powder, boiled and drunk	South Africa	Monakisi, (2007)
*Bersama tysoniana*	hysteria	Barks	Detailed not provided	South Africa	Sobiecki, (2002)
	Male impotence	Stem Bark	Detailed not provided	South Africa	Abdillahi and Van Staden, (2012)
*Bersama stayneri*	Male impotence	Stem Barks	Detailed not provided	South Africa	Abdillahi and Van Staden, (2012)
*Bersama swinnyi*	Male impotence	Barks	Bark decoction or infusion taken orally	South Africa	Abdillahi and Van Staden, (2012)
	Aphrodisiac	Barks	Bark infusion taken orally	South Africa	Hutchings (1996), Ajao et al. (2019)
	Charm and ritual (protection against lightning)	Barks	Detailed not reported	South Africa	Zukulu et al. (2012)
	Headache	Barks	Bark and root infusion taken orally	South Africa	Koorbanally et al. (2008)
	Infertility	Barks	Bark and root decoction or infusion taken orally	South Africa	Hutchings, (1996)
	Leprosy	Barks	Bark and root infusion taken orally	South Africa	Hutchings, (1996)
	Nervous disorders	Barks	Bark and root decoction taken orally	South Africa	Koorbanally et al. (2008)
	Strokes	Barks	Bark and root decoction taken orally	South Africa	Koorbanally et al. (2008)

### 3.2 Phytochemistry

*Bersama* has been thoroughly investigated for its chemical contents, and more than 50 compounds from various chemical classes have been found to date. Steroids, terpenoids, cardiac glycosides, flavonoids, alkaloids, fatty acids, coumarin, xanthonoid, and miscellaneous compounds are found in these phytochemicals. Cardiac glycosides, terpenoids, steroids, flavonoids, and miscellaneous compounds have been discovered as the main components of the majority of *Bersama* genus. The bio efficiency of some of the isolated compounds was also tested. Serial extraction, bioassay-guided extraction, high-performance liquid chromatography (HPLC), apart from successive fractionation using different polarity solvents and column chromatography, Nuclear magnetic resonance (NMR), Electron Ionization-Mass Spectroscopy (EI-MS), Liquid Chromatography-Mass Spectrometry (LC-MS), and Gas Chromatography-Mass Spectrometry (GC-MS) constitute the various methods used to isolate new compounds and elucidate their structures for the *Bersama* genus. Because of the rising demand for traditional medicine as an alternative and supplementary therapy, activity-guided bioactive molecule separation is currently garnering interest (WHO, 2013; Emam et al., 2015). *Bersama* genus are limited in number and mainly found in tropical and subtropical Africa, which explains why so few compounds have been discovered from this genus. This could be due to a variety of circumstances, including plant availability, material constraints, a paucity of skilled labor, and the time-consuming nature of the activity. The summaries for the phytochemical investigation are presented in Table 3.

**TABLE 3:** Compounds isolated from *Bersema* genus.

Compounds	Species	Collection area	Plant organ investigated	Solvent extract	Extraction and identification method used	Ref
Steroids						
Sitosterol 3-*O*-glucopyranose (**1**)	*B. abyssinica*	Kenya	Stem barks	CH_2_Cl_2_	CC, TLC, PTLC, NMR	Ong’era et al. (2017)
β-sitosterol (**2**)	*B. abyssinica*	Kenya	Stem barks	CH_2_Cl_2_	CC, TLC, PTLC, NMR	Ong’era et al. (2017)
	*B. abyssinica*	Ethiopia	Roots	CH_2_Cl_2_/CH_3_OH (1:1)	CC, TLC, NMR	Lemilemu et al. (2020)
	*B. abyssinica*	Uganda	Barks	Petroleum ether	TLC, CC, NMR, MS	Bowen et al. (1985)
7-hydroxy-β-sitosterol (**3**)	*B. abyssinica*	Ethiopia	Roots	CH_2_Cl_2_/CH_3_OH (1:1)	CC, TLC, NMR	Lemilemu et al. (2020)
Stigmasterol (**4**)	*B. abyssinica*	Kenya	Stem barks	CH_2_Cl_2_	CC, TLC, NMR	Ong’era et al. (2017)
Δ^4^-stigmaster-3β-ol (**5**)	*B. engleriana*	Cameroon	Stem Barks	CH_2_Cl_2_	TLC, CC, NMR	Djemgou et al. (2010)
	*B. abyssinica*	Uganda	Barks	Petroleum ether	TLC, CC, NMR, MS	Bowen et al. (1985)
24-propylcholestan-7,15,20-triol (**6**)	*B. swinnyi*	South Africa	Barks	-	TLC, CC, NMR. MS	Koorbanally et al. (2008)
Terepens						
Lupeol (**7**)	*B. abyssinica*	Kenya	Stem barks	CH_2_Cl_2_	CC, TLC, NMR	Ong’era et al. (2017)
	*B. swinnyi*	South Africa	Barks	Chloroform	CC, TLC, NMR	Monkhe et al. (1998)
Betunal (**8**)	*B. swinnyi*	South Africa	Barks	Chloroform	CC, TLC, NMR, MS	Monkhe et al. (1998)
4-methyl-Δ^5-23^-stigmast-dien-3β-ol (9)	*B. abyssinica*	Uganda	Barks	Petroleum ether	TLC, CC, NMR, MS	Bowen et al. (1985)
3-*O*-[β-D-glucopyranosyl-(1 → 2)-β-D-glucuronopyranosyl]-28-*O*-[β-D-glucopyranosyl]-betulinic acid (**10**)	*B. engleriana*	Cameroon	Stem Barks	MeOH/CH_2_Cl_2_ (1:1) and re-extracted with MeOH	TLC, CC, HPTLC, HRESIMS, NMR	Tapondjou et al. (2006)
3-*O*-[β-D-glucopyranosyl-(1 →2)-[β-D-galactopyranosyl-(1→3)]-β-D-glucuronopyranosyl]-oleanolic acid (**11**)	*B. engleriana*	Cameroon	Stem Barks	MeOH/CH_2_Cl_2_ (1:1) and re-extracted with MeOH	TLC, CC, HPTLC, HRESIMS, NMR	Tapondjou et al. (2006)
3-*O*-[β-D-glucopyranosyl-(1 → 3)-β-D-glucuronopyranosyl]-28-*O*-[β-D-xylopyranosyl-(1 →6)-β-D-glucopyranosyl]-oleanolic acid (**12**)	*B. engleriana*	Cameroon	Stem Barks	MeOH/CH_2_Cl_2_ (1:1) and re-extracted with MeOH	TLC, CC, HPTLC, HRESIMS, NMR	Tapondjou et al. (2006)
3-*O*-[β-D-galactopyranosyl-(1 → 3)-b-D-glucuronopyranosyl]-28-*O*-[β-D-glucopyranosyl-(1 →4)-β-D-glucopyranosyl]-oleanolic acid (**13**)	*B. engleriana*	Cameroon	Stem Barks	MeOH/CH_2_Cl_2_ (1:1) and re-extracted with MeOH	TLC, CC, HPTLC, HRESIMS, NMR	Tapondjou et al. (2006)
3-*O*-[β-D-glucopyranosyl-(1 →3)-β-D-galactopyranosyl-(1→3)-b-D-glucuronopyranosyl]-28-*O*-[β-Dxylopyranosyl-(1→6)-β-D-glucopyranosyl]-oleanolic acid (**14**)	*B.engleriana*	Cameroon	Stem Barks	MeOH/CH_2_Cl_2_ (1:1) and re-extracted with MeOH	TLC, CC, HPTLC, HRESIMS, NMR	Tapondjou et al. (2006)
23-Hydroxy betulinaldehyde (swinniol) (**15**)	*B. engleriana*	Cameroon	Stem Barks	CH_2_Cl_2_	TLC, CC, NMR	Djemgou et al. (2010)
	*B. swinnyi*	South Africa	Barks	CHCl_3_	TLC, CC, NMR	Monkhe et al. (1998)
Oleanolic acid (**16**)	*B. swinnyi*	South Africa	Barks	CHCl_3_	TLC, CC, NMR	Monkhe et al. (1998)
4-methylstigmaster-5,23-dien-3β-ol (**17**)	*B. engleriana*	Cameroon	Stem Barks	CH_2_Cl_2_	TLC, CC, NMR	Djemgou et al. (2010)
Lup-30-al-3b-ol (18)	*Bersama lucens*	South Africa	Barks	-	TLC, CC, NMR. MS	Koorbanally et al. (2008)
Lup-20-(30)-en-3,29-diol-7-one (**19**)	*Bersama lucens*	South Africa	Barks	-	TLC, CC, NMR. MS	Koorbanally et al. (2008)
Cardiac Glycosides						
16β-hydroxybersaldegenin-1-acetate (**20**)	*B. abyssinica*	Kenya	Stem barks	Methanol	CC, RPPHPLC, NMR, HRESIMS	Nyamboki et al. (2021)
Paulliniogenin A (**21**)	*B. abyssinica*	Kenya	Stem barks	Methanol	CC, RPPHPLC, NMR, HRESIMS	Nyamboki et al. (2021)
Paulliniogenin B (**22**)	*B. abyssinica*	Kenya	Stem barks	Methanol	CC, RPPHPLC, NMR, HRESIMS	Nyamboki et al. (2021)
16β-formyloxybersamagenin	*B. abyssinica*	Kenya	Stem barks	Methanol	CC, RPPHPLC, NMR, HRESIMS	Nyamboki et al. (2021)
1,3,5-orthoacetate (**23**)						
16β-hydroxybersamagenin	*B. abyssinica*	Kenya	Stem barks	Methanol	CC, RPPHPLC, NMR, HRESIMS	Nyamboki et al. (2021)
1,3,5-orthoacetate (**24**)						
Hellebrigenin 3-acetate (**25**)	*B. abyssinica*	Ethiopia	Stem barks	Ethanol	TLC, CC, NMR	Kupchan et al. (1968)
Hellebrigenin 3,5-diacetate (**26**)	*B. abyssinica*	Ethiopia	Stem barks	Ethanol	TLC, CC, NMR	Kupchan et al. (1968)
Hellebrigenin (**27**)	*B. abyssinica*	Ethiopia	Stem barks	Ethanol	TLC, CC, NMR	Kupchan et al. (1968)
Abyssinin (**28**)	*B. abyssinica*	Ethiopia	Root barks	Methylene chloride	TLC, CC, NMR, EI-MS	Kubo and Matsumoto, (1984)
Tetrahydroabyssinin (**29**)	*B. abyssinica*	Ethiopia	Root barks	Methylene chloride	TLC, CC, NMR, EI-MS	Kubo and Matsumoto, (1984)
3,4-(p-N-,N-dimethylaminobenzoate (30)	*B. abyssinica*	Ethiopia	Root barks	Methylene chloride	TLC, CC, NMR, EI-MS	Kubo and Matsumoto, (1984)
Tetrahydroglycol (**31**)	*B. abyssinica*	Ethiopia	Root barks	Methylene chloride	TLC, CC, NMR, EI-MS	Kubo and Matsumoto, (1984)
17-epiabyssinin (**32**)	*B. abyssinica*	Ethiopia	Root barks	Methylene chloride	TLC, CC, NMR, EI-MS	Kubo and Matsumoto, (1984)
5β, 14β-dihydroxy-3,6-diacetoxy-Δ^6^-bufa-20,22-trienolide (**33**)	*B. abyssinica*	Uganda	Barks	Petroleum ether	TLC, CC, NMR, MS	Bowen et al. (1985)
3-acetoxy-14β-hydroxy-bufa-20,22-dienolide (**34**)	*B. abyssinica*	Uganda	Barks	Petroleum ether	TLC, CC, NMR, MS	Bowen et al. (1985)
Flavonoid						
Hyperoside (**35**)	*B.abyssinica*	Ivory cost, Ethiopia	Leaves	Methanol	TLC, HPLC, HPLC-MS/MS	Asres et al., 2006; Sinan et al., 2021
Vitexin (**36**)	*B. abyssinica*	Ivory cost	Leaves	Methanol	HPLC-MS/MS	Sinan et al. (2021)
Isovitexin (**37**)	*B. abyssinica*	Ivory cost	Leaves	Methanol	HPLC-MS/MS	Sinan et al. (2021)
Morin (**38**)	*B. abyssinica*	Ivory cost	Leaves	Methanol	HPLC-MS/MS	Sinan et al. (2021)
Isoquercetrin (**39**)	*B. abyssinica*	Ethiopia	Leaves	Methanol	TLC, HPLC	Asres et al. (2006)
Quercetin-3-*O*-arabinopyranoside (**40**)	*B. abyssinica*	Ethiopia	Leaves	Methanol	TLC, HPLC	Asres et al. (2006)
Kaempferol-3-*O*-arabinopyranoside (**41**)	*B.abyssinica*	Ethiopia	Leaves	Methanol	TLC, HPLC	Asres et al. (2006)
2,3, 6-trimethoxyflavone (**42**)	*B.abyssinica*	Tanzania	Stem bark and Leaves	Water	LC-MS	Zekeya et al. (2022b)
Alkaloid	*B.abyssinica*	Ivory cost	Leaves	Methanol	HPLC-MS/MS	Sinan et al. (2021)
Kynurenic acid (**43**)	*B. abyssinica*	Ivory cost	Leaves	Methanol	HPLC-MS/MS	Sinan et al. (2021)
Melatonin (44)	*B. abyssinica*	Ivory cost	Leaves	Methanol	HPLC-MS/MS	Sinan et al. (2021)
Picolinos hydrazide (**45**)	*B. engleriana*	Ethiopia	tender foliage	Ethyl acetate	TLC, PTLC,NMR	Ameya et al. (2019)
Xantonoid						
Mangiferin (**46**)	*B. engleriana*	Cameroon	Stem Bark	MeOH/CH2Cl2 (1:1)	TLC, CC, NMR	Djemgou et al. (2010)
	*B.abyssinica*	Ethiopia	Leaves	Methanol	TLC, HPLC	Asres et al. (2006)
	*B. abyssinica*	Kenya	Stem bark	Methanol	CC, RPPHPLC, NMR, HRESIMS	Nyamboki et al. (2021)
Fatty acid						
Azelaic acid (**47**)	*B.abyssinica*	Ivory cost	Leaves	Methanol	HPLC-MS/MS	Sinan et al. (2021)
Eicosanoic acid (**48**)	*B. abyssinica*	Tanzania	Stem bark	Petroleum ether	GC-MS	Zekeya, et al. (2022a)
Linoleic acid (**49**)	*B.abyssinica*	Ivory cost	Leaves	Methanol	HPLC-MS/MS	Sinan et al. (2021)
Coumarin						
7,8-Dihydroxy-4-methylcoumarin (**50**)	*B. abyssinica*	Tanzania	Stem bark and Leaves	Water	LC-MS	Zekeya et al. (2022b)
Miscellaneous Compound						
1,2,3,6-tetra-*O*-galloyl-β-D-glucose (**51**)	*B.abyssinica*	Kenya	Stem bark	Methanol	CC, RPPHPLC, NMR, HRESIMS	Nyamboki et al. (2021)
1,2,6-tri-*O*-galloyl-β-D-glucose (**52**)	*B. abyssinica*	Kenya	Stem bark	Methanol	CC, RPPHPLC, NMR, HRESIMS	Nyamboki et al. (2021)
Methoxyeugenol (**53**)	*B. abyssinica*	Ivory cost	Leaves	Methanol	HPLC-MS/MS	Sinan et al. (2021)
Chlorogenic acid (**54**)	*B. abyssinica*	Ivory cost	Leaves	Methanol	HPLC-MS/MS	Sinan et al. (2021)
Muramic acid (55)	*B.abyssinica*	Ivory cost	Leaves	Methanol	HPLC-MS/MS	Sinan et al. (2021)
2,4-di-tert-butylphenol (**56**)	*B.abyssinica*	Tanzania	Stem bark and Leaves	Water	LC-MS	Zekeya et al. (2022b)
4-formyl-2-methoxyphenyl Propionate (**57**)	*B.abyssinica*	Tanzania	Stem bark and Leaves	Water	LC-MS	Zekeya et al. (2022b)
2-methylamino-butyric acid (**58**)	*B.abyssinica*	Ethiopia	Root	Dichloromethane/Methanol (1:1)	CC, TLC, NMR	Lemilemu et al. (2020)
5-hydroxymethyl-2-furaldehyde (**59**)	*B. abyssinica*	Ivory cost	Leaves	Methanol	HPLC-MS/MS	Sinan et al. (2021)

#### 3.2.1 Steroids

**FIGURE 2:**
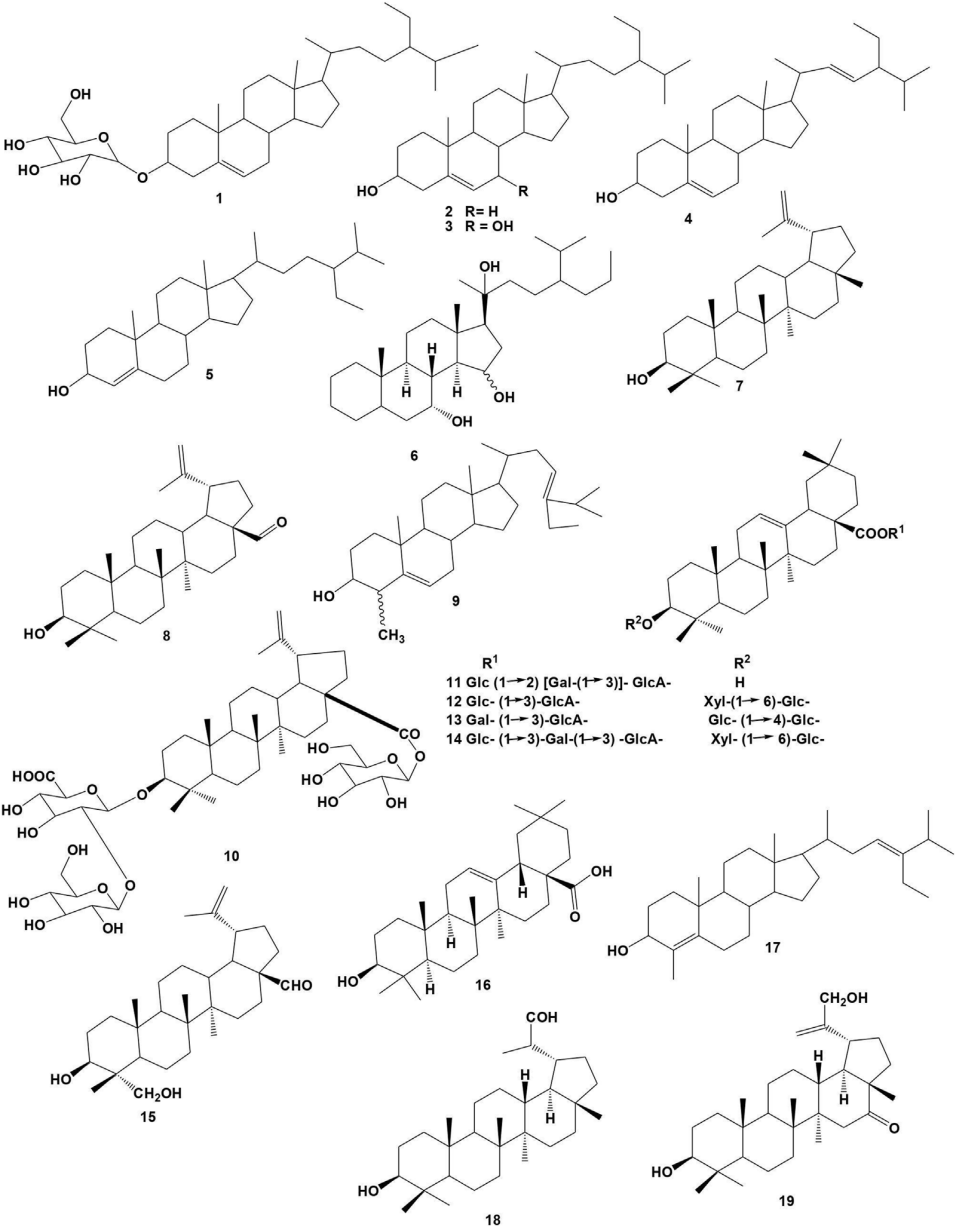
Structure of isolated steroids and terpenoids from *Bersama* genus.

Steroids are complex four-ringed organic molecules that serve many roles and functions in multicellular organisms (Cole et al., 2019). To date, six compounds have been isolated from *Bersama* genus, with the chemical structure presented in Figure 2. Compounds **1**-**4** have been identified from the bark and roots of *B. abyssinica* (Bowen et al., 1985; Lemilemu et al., 2020; Ong’era et al., 2017). Compound **5** was isolated from the bark of both *B. engleriana* and *B. abyssinica* (Bowen et al., 1985; Djemgou et al., 2010) while, compound **6 was** isolated from the bark of *B. swinnyi* (Koorbanally et al., 2008). Only two compounds (**1** and **3**) were evaluated for their pharmacological characteristics. Antibacterial properties of sitosterol-3-*O*-glucopyranose (**1**) and 7-hydroxy-β-sitosterol (**3**) were investigated (Ong’era et al., 2017; Lemilemu et al., 2020).

#### 3.2.2 Terpenes

Terpenes, also known as isoprenoids, are the largest and most diversified group of compounds that are found primarily in plants but also present in animals (Raimundo et al., 2022). They are in charge of plant scent, flavor, and pigment. Terpenes are categorized according to their structure and the number of isoprene units they contain. An isoprene unit is a terpene building block with the chemical formula C_5_H_8_ (Wu et al., 2021). Terpenes and terpenoids are terms that are frequently used interchangeably. Terpenes are natural compounds present in both plants and animals that play a variety of roles in mediating antagonistic and favourable interactions within the organism. Terpene protects many living organisms from abiotic and biotic stresses (Wu et al., 2021). To date, thirteen compounds have been isolated from *Bersama* genus, with the chemical structures presented in Figure 2. Compound **7** was isolated from the stem bark of both *B. abyssinica* and *B. swinnyi* (Monkhe et al., 1998; Ong’era et al., 2017), while compound **8** and **16** were isolated from the bark of *B. swinnyi* (Monkhe et al., 1998). Compound **9** was identified from the bark of *B. abyssinica* (Bowen et al., 1985). Compounds **10**-**14** were isolated from *B. engleriana* barks (Tapondjou et al., 2006; Djemgou et al., 2010). Compound **15** was isolated from both *B. engleriana* and *B. swinnyi* (Monkhe et al., 1998; Djemgou et al., 2010). Compounds 18 and 19 were isolated from *B. lucens* (Koorbanally et al., 2008), while compound 17 was isolated from the stem bark of *B. engleriana* (Djemgou et al., 2010). Only one compound, lupeol (**7**), was evaluated for its pharmacological characteristics, including antibacterial properties. (Ong’era et al., 2017).

#### 3.2.3 Cardiac glycosides

**FIGURE 3:**
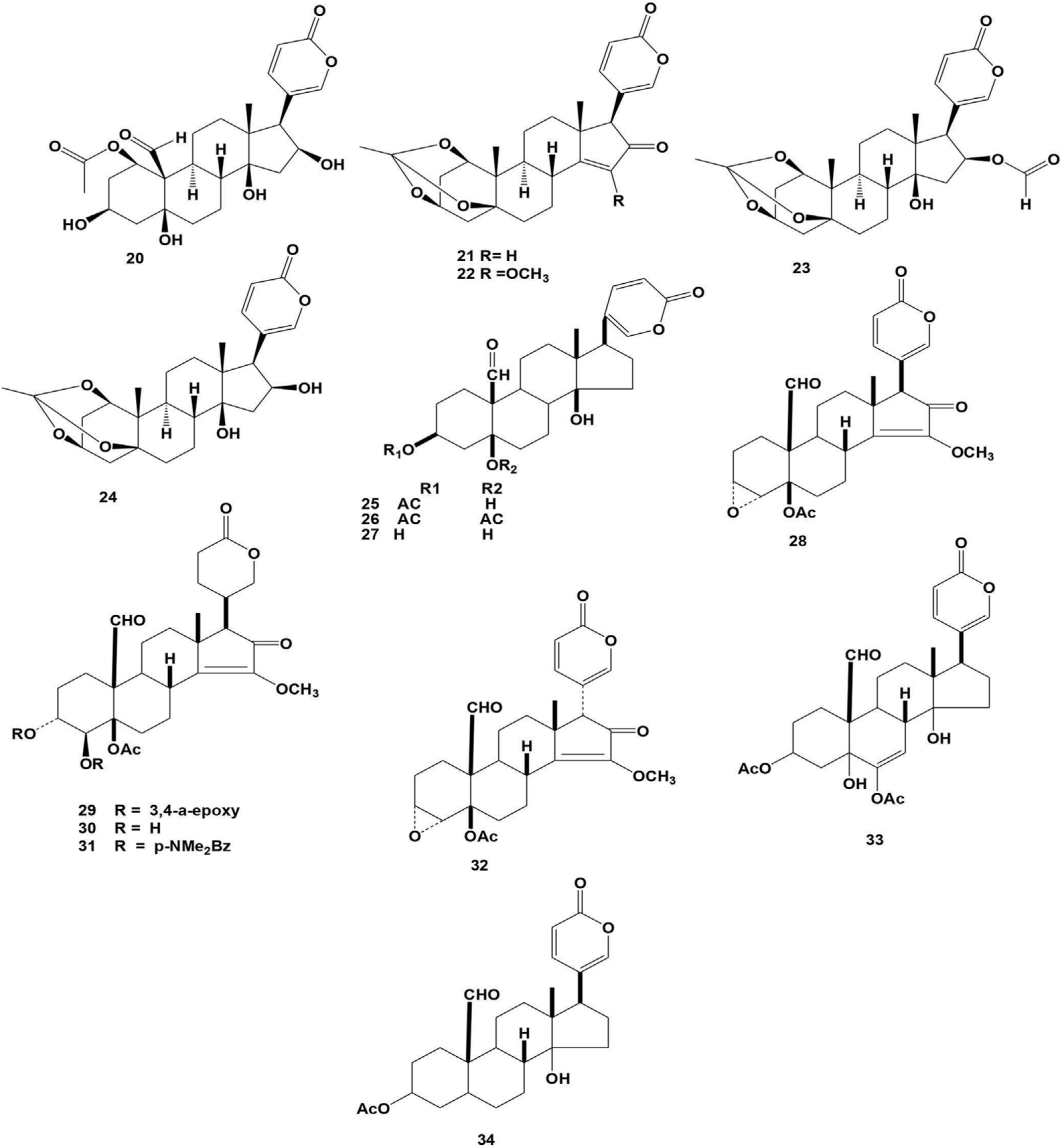
Structure of isolated cardiac glycosides from *Bersama* genus.

Cardiac glycosides are a diverse class of naturally occurring compounds that bind to and inhibit the Na+/K + -ATPase enzyme. Members of this family have been used in clinical trials for many years to treat heart failure and atrial arrhythmia, and the mechanism behind their positive inotropic action is well known. Recent discoveries have proposed new signaling mechanisms of action for Na+/K + -ATPase, involving cardiac glycosides in the control of numerous essential cellular processes and revealing possible new therapeutic functions for these compounds in a variety of disorders (Prassas and Diamandis, 2008; Škubník et al., 2021). The enhanced vulnerability of cancer cells to these compounds, perhaps most notably, indicates their prospective application as cancer therapeutics, and the first generation of glycoside-based anticancer medicines is now in clinical trials (Calderón-Montaño et al., 2014). To date, 14 compounds had been isolated from the *Bersama* genus and their chemical structures were depicted in Figure 3. Compounds from **20-34** were isolated from the stem bark, root bark and bark of *B. abyssinica* (Kupchan et al., 1968; Kubo and Matsumoto, 1984; Bowen et al., 1985; Nyamboki et al., 2021). Only four compounds (**21**, **23**, **25**, and **26**) were evaluated for their pharmacological properties. The cytotoxicity of paulliniogenin A (**21**), 16-formyloxybersamagenin-1,3,5-orthoacetate (**23**), hellebrigenin-3-acetate (**25**), and hellebrigenin-3,5-diacetate (**26**) was investigated (Kupchan et al., 1968; Nyamboki et al., 2021).

#### 3.2.4 Flavonoid

**FIGURE 4:**
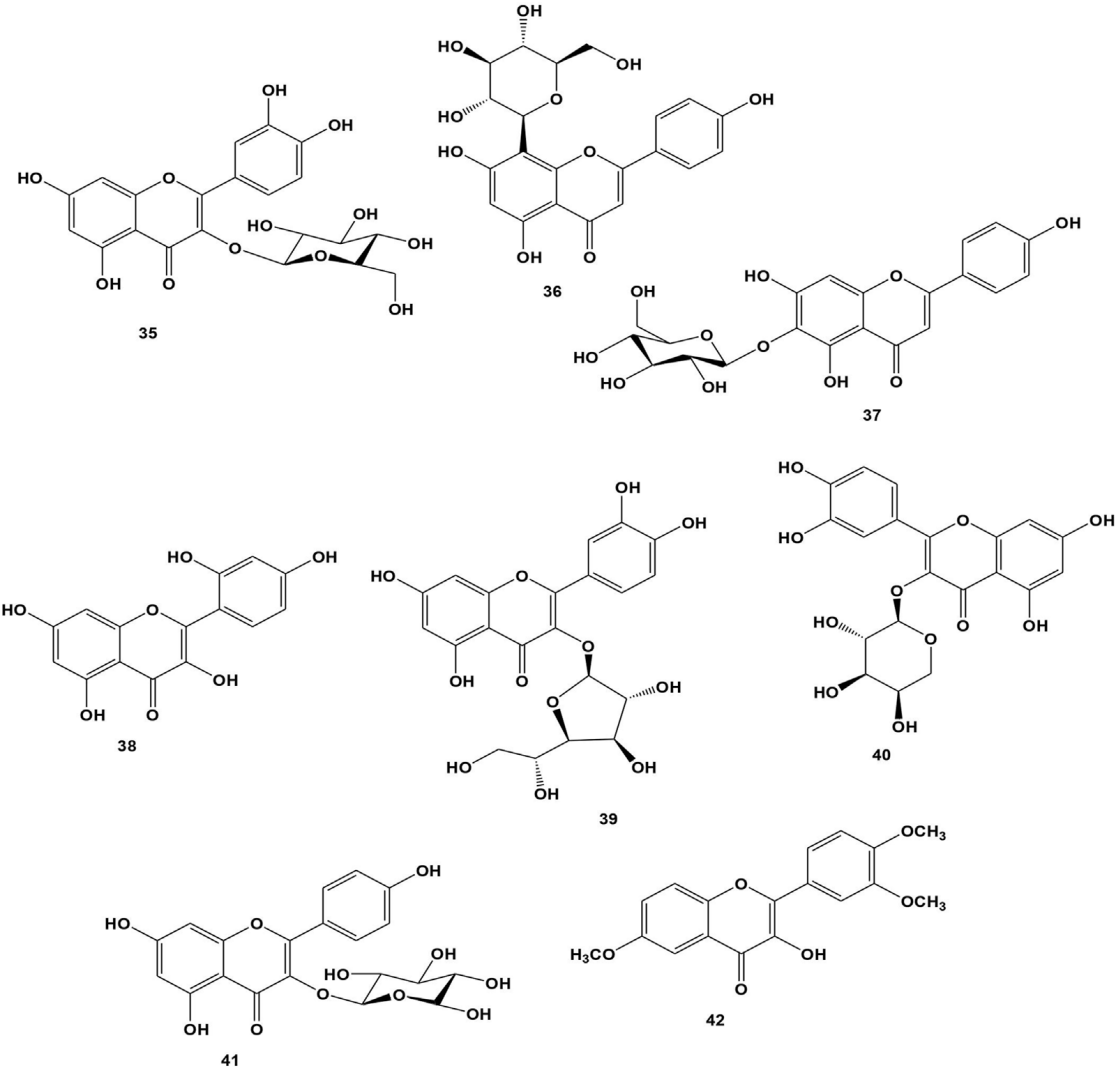
Structure of isolated flavonoids from *Bersama* genus.

Flavonoids are hydroxylated phenolic compounds that plants produce in response to microbial infection (Kumar and Pandey, 2013). Numerous flavonoids have been found to have antioxidative, free radical scavenging, hepatoprotective, anti-inflammatory, and anticancer properties, and certain flavonoids also show potential antiviral properties. Flavonoids serve as growth regulators and assist plants to resist oxidative stress. Microbial biotechnology has enabled the cost-effective bulk manufacturing of many types of flavonoids for therapeutic uses (Yonekura-Sakakibara et al., 2019). Eight compounds have been isolated to date from *B. abyssinica* where compounds, **35**-**42**, were isolated from the leaves (Asres et al., 2006; Sinan et al., 2021; Zekeya et al., 2022a). Using the DPPH assay and quercetin as a positive control, the antioxidant activity of compounds **39**, **35**, and **40** were examined. When compared to quercetin (18.2 μM), the compounds isoquercetin (**39**), hyperoside (**35**), and quercetin-3-*O*-arabinopyranoside (**40**) showed IC_50_ values of 23.7, 22.6, and 20.7 μM, which are promising (Asres et al., 2006). The chemical structures are presented in Figure 4.

#### 3.2.5 Alkaloid

**FIGURE 5:**
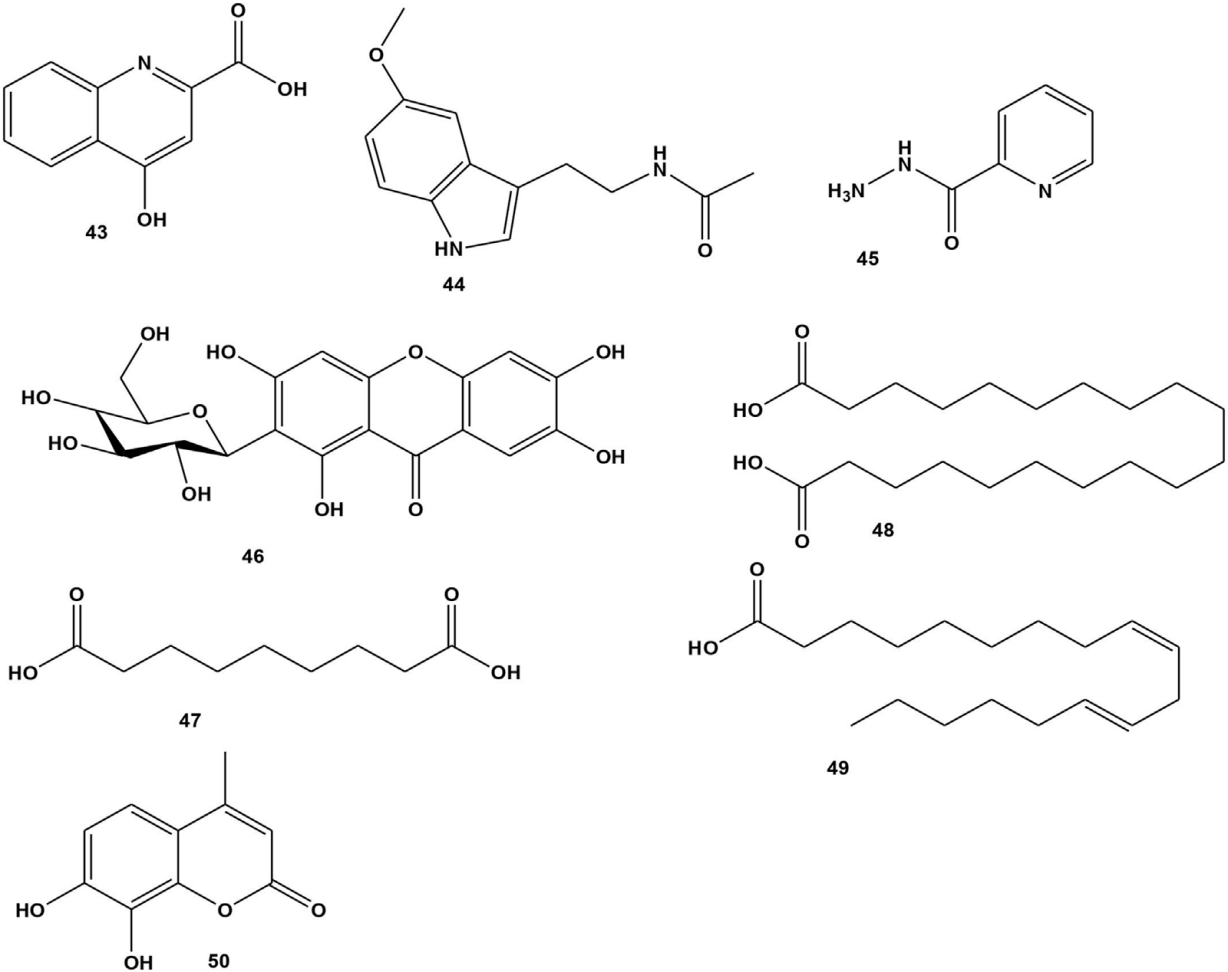
Structure of isolated alkaloids, xanthonoid, fatty acid and coumarin from *Bersama* genus.

Alkaloids are well-known nitrogen-containing natural chemical compounds with a wide range of pharmacological activities including antibacterial, antioxidant, cytotoxicity, and anti-inflammatory properties (Kukula-Koch and Widelski, 2017; Debnath et al., 2018). So far, two compounds **43** and **44** have been isolated from *B. abyssinica* leaves, while one compound **45** was identified from *B. engleriana* tender foliage (Ameya et al., 2019; Sinan et al., 2021). The chemical structures are shown in Figure 5.

#### 3.2.6 Xanthonoid

A xanthonoid is a phenolic natural chemical compound derived from the xanthone backbone. Mangiferin is a typical C-glycosyl xanthone compound with numerous pharmacological properties (Matkowski et al., 2013). The mangiferin molecule’s powerful antiradical and antioxidant properties are governed by four aromatic hydroxyl groups. Mangiferin is also an excellent iron chelator, inhibiting the production of hydroxyl radicals in Fenton-type reactions. Many other analgesic, antidiabetic, antibacterial, antiviral, cardioprotective, hepatoprotective-, neuroprotective, anti-inflammatory and antiallergic properties of mangiferin have been reported in multiple published *in vitro* and *in vivo* pharmacological studies (Matkowski et al., 2013). Mangiferin (**46**) was the only chemical compound isolated from *B. abyssinica* leaves and stem barks and *B. engleriana* stem barks (Asres et al., 2006; Djemgou et al., 2010; Nyamboki et al., 2021). The compound’s antioxidant properties were evaluated using the DPPH method using quercetin as a positive control, and showed higher activity when compared to the positive control, with IC_50_ values of 15.9 and 18.2 μM for mangiferin and positive control, respectively (Asres et al., 2006). Its chemical structure is presented in Figure 5.

#### 3.2.7 Fatty acid

Fatty acids are composed of hydrocarbon chains that end in carboxylic acid groups. Fatty acids and their derivatives are the primary components of lipids (Lee et al., 2016). Fatty acids, in the form of triacylglycerol, play key roles in signaling pathways, cellular fuel sources, hormone and lipid composition, protein modification, and energy storage inside the adipose tissue (Calder, 2015). Three compounds have been isolated from the leaves and stem barks of *B. abyssinica*: azelaic acid (**47**), eicosanoic acid (**48**), and linoleic acid (**49**) (Sinan et al., 2021; Zekeya et al., 2022a). The chemical structures presented in Figure 5.

#### 3.2.8 Coumarin

Coumarins are phenolic compounds made up of fused benzene and α-pyrone rings. The coumarin structure is formed from cinnamic acid via ortho-hydroxylation, trans-cis isomerization of the side-chain double bond, and lactonization (Venugopala et al., 2013). Coumarins contain antithrombotic, anti-inflammatory, antioxidant, anticancer and vasodilatory properties (Küpeli Akkol et al., 2020). One compound, 7-8-dihydroxy-4-methyl coumarin (**50**), was isolated from the stem bark of *B. abyssinica* (Zekeya et al., 2022b). Its chemical structure is presented in Figure 5.

#### 3.2.9 Miscellaneous compounds

**FIGURE 6:**
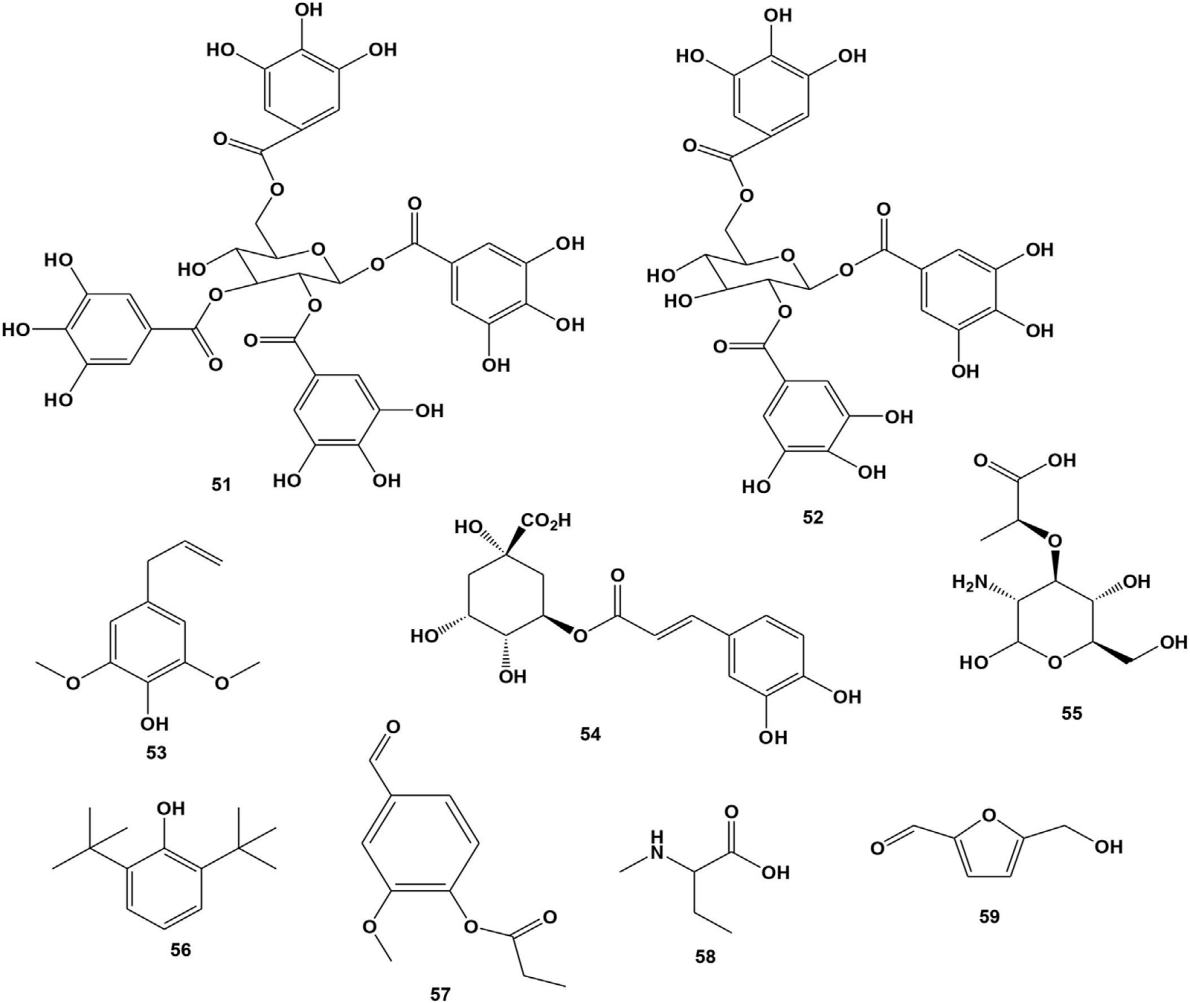
Structure of isolated miscellaneous compounds from *Bersama* genus.

So far, nine compounds (**51**-**59**) were isolated from the stem bark, leaves and roots of *B. abyssinica* (Nyamboki et al., 2021; Sinan et al., 2021; Zekeya et al., 2022a). The biological actions of these compounds are not yet revealed. Their chemical structures are shown in Figure 6.

### 3.3 Pharmacological activities

**FIGURE 7:**
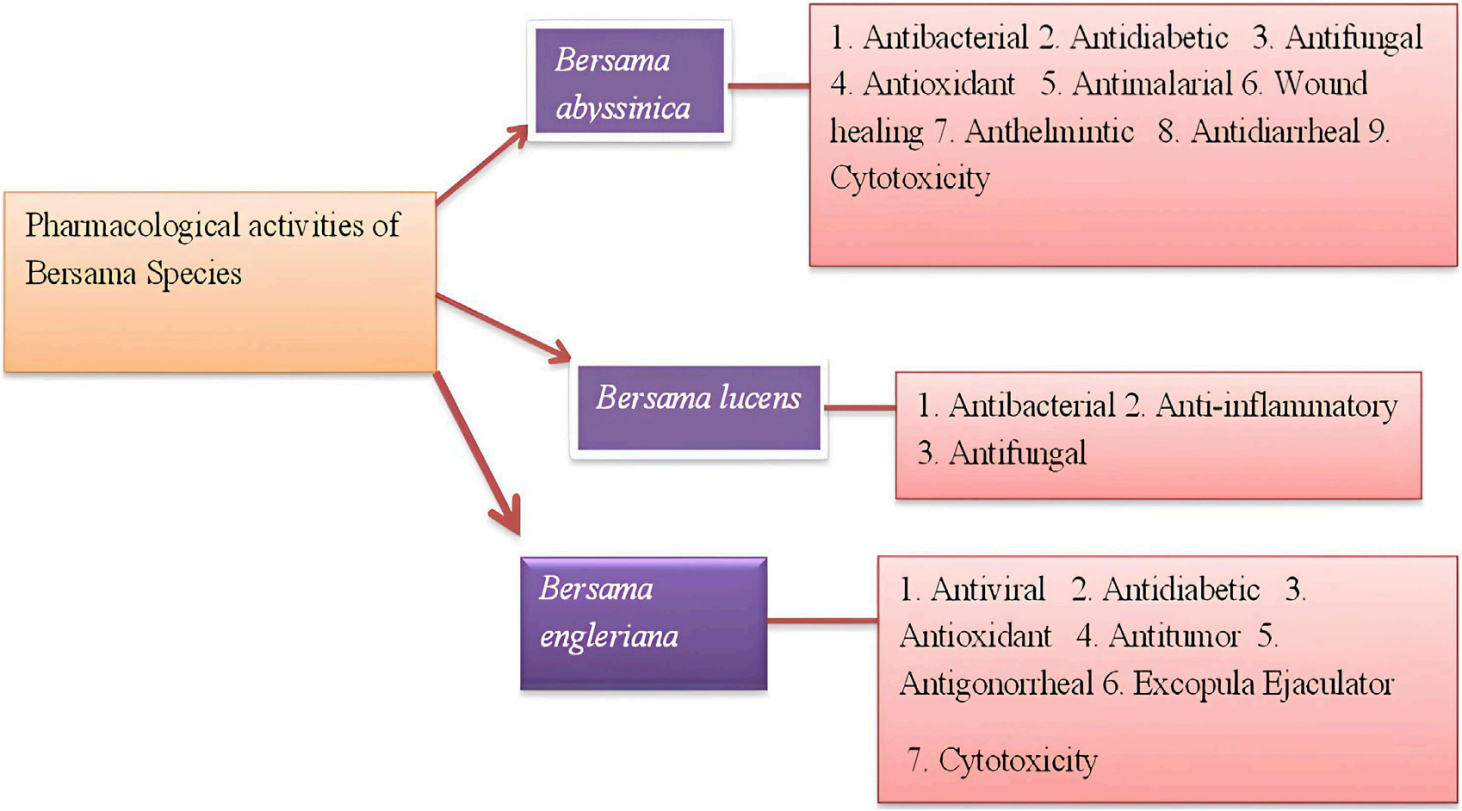
Reported pharmacological activities of *Bersama* genus.

Modern and traditional methods of healthcare frequently coexist and complement one another. Ethnomedicinal practices are currently routinely used in the search for innovative medications (Yuan et al., 2016). Recently, there has been increasing interest in investigating plant components for pharmacological activity and screening for beneficial and safe phytochemicals (Nigussie and Wale, 2022). In traditional medicine, *Bersama* genus have been used to treat a variety of illnesses, including leprosy, stroke, diabetes, hepatitis, cancer, impotency, infertility, cancer and utilized as psychotropic medications and blood purifier (Table 2). Antibacterial, antifungal, antimalarial, antioxidant, anthelmintic, antiviral, antimalarial, antidiabetic, antitumor, and cytotoxic effects of *Bersama* genus are shown in Figure 7 and which are summarized in Supplementary Table S1.

#### 3.3.1 Antibacterial activities

The antibacterial activity of the *B. abyssinica* methanol stem bark extracts was evaluated using the disc diffusion method and ampicillin as a positive control against *Bacillus subtilis* (*B. subtilis*), *Escherichia coli* (*E. coli*), *K. pneumoniae* (*Klebsiella pneumoniae*), *Pseudomonas aeruginosa* (*P. aeruginosa*), *Staphylococcus aureus (S. aureus)*, and *V. cholerae* (*V.cholerae*). In comparison to the inhibition zone of ampicillin, which range from 21 to 23 mm, *K. pneumonia* and *P. aeruginosa* both had highest inhibition zones of 16 mm each (Ong’era et al., 2017). The compound sitosterol 3-*O*-glucopyranose (**1**), isolated from dichloromethane stem bark extracts of *B. abyssinica,* exhibited moderate activity against *S. aureus* with inhibition zones of 14 and 13 mm for *K. pneumoniae*, respectively, and inhibition zones of 10 and 15 mm for *V. cholerae* and *E. coli* (Ong’era et al., 2017). Similarly, lupeol (**7**) displayed modest activity against *S. aureus*, with an inhibition zone of 14 mm and it also had inhibition zones of 11 and 8 mm against *B. subtilis* and *E. coli*, respectively (Ong’era et al., 2017). The antibacterial activity of *B. abyssinica* DCM/MeOH (1:1) and MeOH root extracts against *E. coli*, *S. thyphimerium*, *S. aureus*, and *B. subtlis* was investigated using disc diffusion assay with ciprofloxacin as positive control. The extracts were less active in both solvent systems, with an inhibition zone ranging from 11 to 13.6 mm, than the standard drug, which had an inhibition zone ranging from 26 to 34 mm. The compound 7-hydroxy-β-sitosterol (**3**) showed promising antibacterial activity against *E. coli* and *S. aureus*, with zones of inhibition of 12.6 and 12.5 mm, respectively (Lemilemu et al., 2020). The methanol leaves extracts of *B. abyssinica* were tested against *Xanthomonas campestris pv musacearum* bacteria with a MIC of 25 mg/mL, which is less than that of the standard drug tetracycline, which has a MIC of 0.02 mg/mL (Yemata et al., 2019). The antibacterial activity of *B. abyssinica* ethanol root extracts were investigated against *Salmonella typhimurium* (ATCC 14028), *Salmonella typhi* and *P. aeruginosa* using ciprofloxacin as a positive control. The extract exhibited MIC values of 10, 10 and 2.5 mg/mL respectively, which is modest activity when compared to ciprofloxacin’s MIC value of 0.025 mg/mL (Bolou et al., 2011). The antibacterial properties of tender foliage ethyl acetate extracts of *B. abyssinica* were investigated against MDR Gram-negative and positive pathogen bacteria *E. coli*, *K. pneumonia*, *S. aureus*, and *E. faecalis*. The extract showed MIC values ranged from 12.5 to 100 mg/mL (Ameya et al., 2019). The antimicobacterial effects of *B. abyssinica* methanol leaves stem bark and root bark extracts against *Mycobacteria madagascariense* and *Mycobacteria indicuspranii* were investigated using rifampicin as a positive control. The extracts’ MIC values varied from 0.19 to 0.78 mg/mL, which is more comparable to the standard rifampicin’s MIC value of 0.19 mg/mL (Mwambela et al., 2014). Buwa and Van Staden (2006) evaluated the antibacterial activities of water, ethyl acetate and ethanol extracts of *B. lucens* bark against *K. pneumoniae* ATCC 13883, *B. subtilis* ATCC 6051, *S. aureus* ATCC 12600 and *E. coli* ATCC 11775 using the micro plate method with neomycin as a positive control. The water and ethanol extracts exhibited activities against tested pathogens with minimum inhibitory concentration (MIC) values ranging from 3.1 mg/mL to >12.5 mg/mL. Khumalo (2018) evaluated the antibacterial activities of dichloromethane and methanol extracts of *B. lucens* bark against *Bacillus cereus* ATCC 11175, *Enterococcus faecalis* ATCC 29121, *E. coli* ATCC 8739, *S. typhimurium* ATCC 14028 and *Shigella sonnei* ATCC 9290 using the micro-titer plate technique with ciprofloxacin as a positive control. The extracts exhibited activities with MIC values ranging from 0.3 mg/mL to 2.0 mg/mL in comparison to MIC values of 0.02 μg/mL to 0.07 μg/mL exhibited by the positive control (Khumalo, 2018).

#### 3.3.2 Antiviral activities

Using HIV-1 (IIIB) and HIV-2 (ROD) strains, the methanol root extract of *B. abyssinica* was tested for its ability to inhibit viral replication. The simultaneous assessment of the extracts’ *in vitro* cytotoxicity against MT-4 cells using MTT assay allowed for the evaluation of the selective inhibition of viral growth. The methanol extracts from the root bark of *B. abyssinica* inhibited HIV-1 replication at 50% effective concentrations (EC_50_) of 3.1 mg/mL with a corresponding selectivity index of 3.8 (Asres et al., 2001). The anti-reverse transcriptase activity of *Bersama engleriana’s* leaves, barks, and roots was examined using recombinant HIV-enzyme, a non-radioactive HIV-RT colorimetric ELISA kit, and doxorubicin (100 μg/mL) as a positive control. The extracts’ respective IC_50_ values of 11.95, 18.75, and 9.38 μg/mL indicated moderate activity in comparison to the positive control drug doxorubicin, whose IC_50_ value was 4.24 μg/mL (Mbaveng et al., 2011). The antiviral efficacy of *B. abyssinica* water extract stem bark was tested against the SARS-CoV-2 virus DELTA strain (BS-01). At 16 and 50 μg/mL concentrations, the extract exhibited the best inhibitory action against Delta B1, producing 75% virus mortality with negligible cytotoxicity effects on host cells (Zekeya et al., 2022b).

#### 3.3.3 Antidiabetic activities

The anti-diabetic properties of *B. engleriana* leaves were evaluated in nicotinamide/STZ-induced diabetic adult male Wistar rats using glibenclamide as a standard drug. The methanol extracts suppressed blood glucose concentrations by 80.31% at a dose of 600 mg/kg, which was more effective than the standard drug glibenclamide (58.65%) (Pierre et al., 2012). The α-amylase inhibitory activities of 80% methanol extracts of *B. abyssinica* leaves were determined using the 3,5-dinitrosalicylic acid method and acarbose as a positive control. The crude extract inhibited α-amylase enzyme with an IC_50_ of 6.57 μg/mL, which is comparable to acarbose’s IC_50_ of 2.26 μg/mL. The extracts’ blood glucose lowering activity was also investigated in four animal models: normoglycemic, oral glucose loaded, and streptozotocin-induced diabetic mice. All doses (100,200, and 400 mg/kg) of the crude extract significantly (P˂ 0.05) reduced blood glucose levels in oral glucose-loaded and streptozotocin-induced diabetic mice models (Kifle and Enyew, 2020). The anti-diabetic properties of *B. engleriana* methanol leaves extract in streptozotocin/nicotinamide (STZ-NA)-induced type 2 diabetic rats were evaluated. The extracts at doses of 300 or 600 mg/kg were administered orally to animals for 4 weeks. Blood glucose (BG) levels were measured at 0, 1, 14, and 28 days after STZ-NA treatment, as well as total cholesterol (TC), high density lipoprotein cholesterol (HDL-C), low density lipoprotein cholesterol (LDL-C), and triglycerides (TG) levels at sacrifice (day 29). When compared to controls, STZ-NA-induced diabetic rats had moderate to significant increases in BG, TG, TC, and LDL-C levels, but body weight, HDL-C levels, and relative weights of liver and pancreas were decreased (non-diabetic rats). The methanolic extract at 600 mg/kg showed to be the most effective; HDL-C levels were significantly higher after 4 weeks compared to untreated diabetic rats, and the effects were greater (p˂ 0.001) than glibenclamide (0.25 mg/kg) (Pierre et al., 2012). The hypoglycemic properties of *B. abyssinica* leaves solvent fractions (aqueous and ethyl acetate) were evaluated using normoglycemic mice and glibenclamide as a positive control. The percentage reduction in baseline blood glucose levels was 25.90%, 26.36%, 38.43%, 30.96%, and 49.42% for EAF200 mg/kg, AQF200 mg/kg, EAF400 mg/kg, AQF400 mg/kg, and GLC 5 mg/kg, respectively (Kifle et al., 2020).

#### 3.3.4 Antifungal activities

Aqueous and ethanol extracts of the leaf of *B. abyssinica* were investigated for antifungal activity against *Aspergillus flavus*, which produces aflatoxin B1. The extracts exhibited MIC values of 98 and 195 μg/mL, respectively (Bene et al., 2017). The antifungal properties of *B. abyssinica* leaves, stem, bark, and root extracts against the coffee pathogenic fungus *Gibberella xylarioides* were examined. The extracts have MIC values of 0.19, 0.78, and 0.78 mg/mL (Mwambela and Kilambo, 2011). The antifungal properties of *B. engleriana* methanolic roots, stem barks, leaves, and wood were examined against *Candida albicans* and *Candida gabrata* using an agar diffusion assay and nystatin as a control drug. The extracts had MIC values ranging from 9.76 to 39.06 μg/mL and MBC values ranging from 19.53 to 78.12 μg/mL, which were less potent than the positive control, which had MIC and MBC values of 2.44 and 4.88 μg/mL, respectively (Kuete et al., 2008). Buwa and Van Staden (2006) evaluated the antifungal activities of water, ethyl acetate and ethanol extracts of *B. lucens* bark against *C. albicans* ATCC 10231 using the micro plate method with neomycin as a positive control. The extracts exhibited activities against the tested pathogen with MIC values ranging from 0.78 mg/mL to 12.5 mg/mL.

#### 3.3.5 Antioxidant activities

**FIGURE 8:**
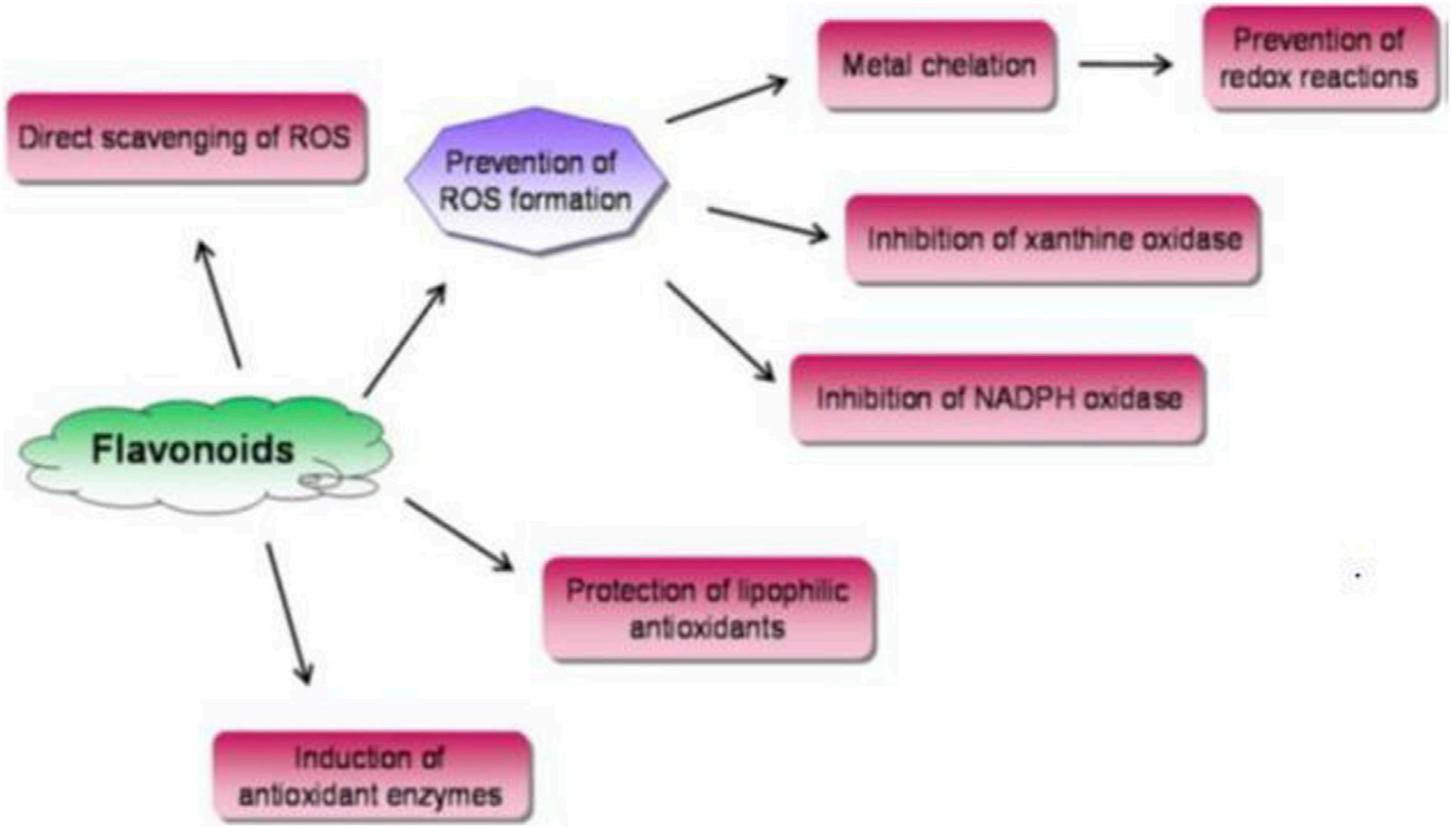
Mechanisms of antioxidant effects of flavonoids.

The antioxidant properties of *B. abyssinica* 80% methanol leaves extracts and aqueous fraction were evaluated using the DPPH method with ascorbic acid as a positive control. The IC_50_ values for the crude extract and fraction were 5.35 and 3.43 μg/mL, respectively, which are comparable to the standard ascorbic acid IC_50_ value of 2.65 μg/mL (Kifle and Enyew, 2020). The antioxidant activities of methanolic extracts of *B. engleriana* root, stem bark, leaves, and wood were investigated using the DPPH method. The DPPH• scavenging activity revealed that the extract from the leaves was the most active, with a 93.71% inhibition rate at 1,000 μg/mL (Kuete et al., 2008). The antioxidant properties of *B. abyssinica* methanol leaves extracts were evaluated using the DPPH method with quercetin as a positive control. The extract has an IC_50_ value of 7.5 μg/mL, which is considered moderate activity when compared to the positive control quercetin, which had an IC_50_ value of 18.2 μM (Asres et al., 2006). The antioxidant properties of the identified compounds isoquercetrin (**39**), hyperoside (**35**), quercetin-3-*O*-arabinopyranoside (**40**), and mangiferin (**46**) from methanol extracts of *B. abyssinica* were also examined using the DPPH assay. The compounds had IC_50_ values of 23.7, 22.6, 20.7, and 15.9 μM, which are promising when compared to quercetin (Asres et al., 2006). Compounds (39, 35, and 40) are flavonoids, which are abundant naturally occurring phenolic compounds well known for their antioxidant properties they may exert their antioxidant effects via the mechanism action of preventing ROS generation, direct scavenging of ROS, or indirectly through enhancement of cellular antioxidant enzymes (Santos-Sánchez et al., 2019; Xu H. et al., 2024) (Figure 8). These compound capabilities are linked to a lower risk of neurological illnesses such as cardiovascular disease, gastrointestinal cancers, colon, breast, and ovarian cancers, as well as leukemia (Olszowy M., 2019). Mangiferin is a xanthonoid compound that is used in treatment to lower AGE development and xanthine oxidase activity. Xanthine oxidase and advanced glycation end products (AGE) are involved in ROS formation; hence, mangiferin reduces ROS levels and oxidative damage, allowing for the recovery of sepsis-related organ damage (Rahmani et al., 2023).

#### 3.3.6 Antitumor activity

The antitumor activity of methanolic extracts of *B. engleriana* root, stem bark, leaves, and wood were examined using crown gall tumor tests. Extracts from the roots (69.32%) and leaves (65.42%) exhibited considerable tumor-reducing efficacy (Kuete et al., 2008).

#### 3.3.7 Antimalarial activities

The antimalarial properties of 80% methanol leaves extract and fractions (aqueous, ethyl acetate, and chloroform) of *B. abyssinica* in *Plasmodium berghei* ANKA infected mice and chlroquine as reference drug were investigated. In comparison to the negative control, the 80% methanolic crude extract and all solvent fractions of *B. abyssinica* leaves demonstrated statistically significant (*p* < 0.05 to *p* < 0.001) chemosuppressive efficacy against *P. berghei* infection in mice. The crude extract (49.51%, *p* < 0.001), aqueous (47.69%, *p* < 0.001), ethyl acetate (41.89%, *p* < 0.001), and chloroform (38.21%, *p* < 0.001) exhibited the highest chemosuppression at 400 mg/kg dose, which was less than the standard drug, chloroquine (25 mg/kg), showed 100% chemosuppression. The crude extracts at 200 and 400 mg/kg doses significantly increased the mean survival time of mice (10.50 ± 0.76 days and 13.83 ± 1.05 days, respectively, *p* < 0.001) when compared to the negative control (6.17 ± 0.40) (Alehegn et al., 2020). Using chloroquine as a positive control, the antimalarial effects of *B. abyssinica* ethanol leaves extract were examined against a strain of *Plasmodium falciparum* that was resistant to chloroquine. The extract’s IC_50_ value was 23.9 μg/mL, which is less powerful than the IC_50_ value of 0.1 μg/mL for the positive control (Zirihi et al., 2005). The antiplasmodial properties of *B. abyssinica* extracts in dichloromethane/methanol (1:1) were examined against *P. falciparum* W_2_ strains (chloroquine resistance) and D_6_ strains (chloroquine sensitivity) using a semi-automated micro-dilution technique, with chloroquine used as a positive control. The extract had an IC_50_ of 12.85 and 8.48 μg/mL against D_6_ and W_2_ strains, respectively, which was less potent than the positive control chlroquine, which had IC_50_ values of 0.00124 and 0.00153 μg/mL against D_6_ and W_2_ strains, respectively (Omole et al., 2020). The antispasmodic effect of *B. abyssinica* aqueous leaves extract was investigated using Guinea pig ileum with isotonic contractions and different concentrations of a standard spasmogenic and histamine. The extract was found to antagonize the spasmogenic effect of histamine in a nonreversible manner (Makonnen and Hagos, 1993).

#### 3.3.8 Anti-gonorrheal activities

Using the dilution method and gentamicin as a positive control, the anti-gonorrhoeic activity of *B. engleriana* bark methanol extract was tested against ATCC 49226, β-lactamase negative WHO (A) and (B), clinical β-lactamase negative (NGCS1-4) and β-lactamase positive (NGCS5-7) of *Neisseria gonorrhoeae*. The extract shown substantial activity against ATCC 49226, WHO A (βL−), *NGCS*1 (βL−), *NGCS*3 (βL−), NGCS5 (βL +) and *NGCS*5 (βL +) with MIC values of 16 μg/mL; gentamicin’s MIC values ranged from 0.5 to 32 μg/mL (Mbaveng et al., 2011).

#### 3.3.9 Wound healing activity

Wound healing properties of 80% hydro-methanol leaves extracts of *B. abyssinica* at 5% and 10% w/w ointment were investigated in excision, incision, and burn wound models using simple ointment and nitrofurazone 0.2% w/v as positive control. On the excision wound healing model, the extract produced 5% (99.5%) and 10% (100%) wound contraction on the 16^th^ day of treatment, as well as 5% (18.8) and 10% (28.2) reduction in epithelization, which is comparable to the positive control nitrofurazone, which produced 100% wound contraction and 27.4% reduction in epithelization (Taddese et al., 2021).

#### 3.3.10 Anthelmintic activity

The anthelmintic effects of *B. abyssinica* methanol and ethanol extracts of the leaves on *Haemonchus contortus* were investigated using an egg hatch assay and alebendazole as a control drug. When compared to alebendazole (0.005 mg/mL), which indicated 99.33% and 99.66% action, the mean percentage suppression of *H. contortus* egg hatching after 48 h exposure at 2 mg/mL was 95.67% and 89% at the same concentration, respectively (Asian, 2018).

#### 3.3.11 Antidiarrheal activity

The antidiarrheal activity of crude extracts and solvent fractions of *B. abyssinica* leaves was investigated in mice using castor oil-induced diarrhea, enteropooling, and antimotility tests, with loperamide 3 mg/kg and 2% Tween 80 used as positive and negative controls. At dosages of 100, 200, and 400 mg/kg of crude extract and aqueous fraction, defecation of castor oil-induced diarrheal or loose stools was inhibited (*p* < 0.01 to *p* < 0.001). The crude extract and aqueous fraction at three doses (*p* < 0.01 to *p* < 0.001), the chloroform fraction at 200 mg/kg and 400 mg/kg (p ˂ 0.01 to p ˂ 0.001), and the n-hexane fraction at 400 mg/kg (p ˂ 0.05) all reduced intraluminal fluid accumulation when compared to the negative control. The crude extract (70.83%) significantly suppressed Castor oil-induced intestinal motility by 70.83%, which is comparable to the positive control loperamide (75.0%) at 400 mg/kg (Ayalew et al., 2022).

#### 3.3.12 Anti-inflammatory activities

McGaw et al. (1997) evaluated the anti-inflammatory activities of the aqueous and ethanol leaves extracts of *B. lucens* by assessing the inhibition of prostaglandin biosynthesis using the cyclooxygenase assay with indomethacin (0.5 μg) as a positive control. The extracts exhibited suitable activities with percentage inhibition ranging from 71.0% to 80.0%, which were higher than 75.0% exhibited by the positive control (McGaw et al., 1997).

#### 3.3.13 Anticancer activity

**FIGURE 9:**
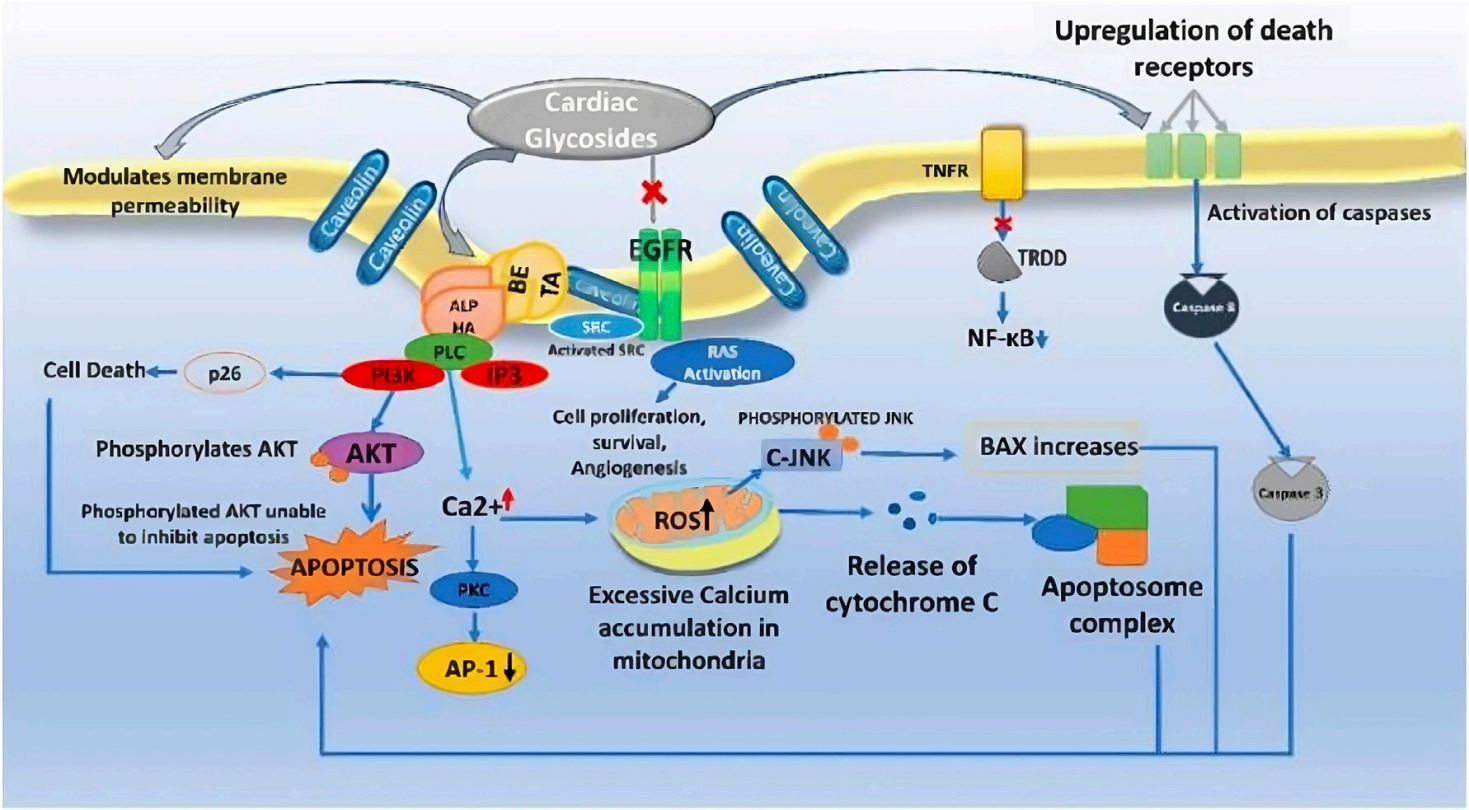
Anticancer mechanism of cardiac glycosides.

The cytotoxic effects of *B. abyssinica* ethanol stem ark extracts were evaluated using the brine shrimp method and cyclophosphamide as a reference drug. The extract had an LC_50_ value of 7.8 μg/mL, which was higher than the standard drug’s LC_50_ value of 16.3 μg/mL (Moshi et al., 2010). The cytotoxicity of compounds paulliniogenin A (**21**) and 16β-formyloxybersamagenin-1,3,5-orthoacetate (**23**) obtained from the stem bark methanol extract of *B. abyssinica* sub species was evaluated against mammalian cell lines HeLa (KB3.1) using MTT assay and epithilon as positive control. Paulliniogenin A (**21**) and 16β-formyloxybersamagenin-1,3,5-orthoacetate (**23**) demonstrated cytotoxicity against the KB3.1 cell line with IC_50_ values of 1.4 and 1.6 μM, respectively, which were less potent than the standard drug, which had an IC_50_ value of 0.000056 μM (Nyamboki et al., 2021). The cytotoxic activity of *B. abyssinica* stem bark methanol extracts was examined using the brine shrimp larvae method with cyclophosphamide as a positive control. The extract showed an LC_50_ value of 29.64 μg/mL, which was less than the standard drug’s LC_50_ value of 16.4 μg/mL (Mwambela et al., 2014). The cytotoxicity activities of *B. engleriana* leaves methanol extracts were examined against cell lines THP-1, DU145, HeLa, MCF-7, and HepG_2_ using the XTT assay with doxorubicin as a positive control. The extract had IC_50_ values of 100, 15.7, 50.8, 8.6, and 20.3 μg/mL respectively, indicating that the methanol leaves extract is more effective in DU145, MCF-7, and HepG_2_, when compared to doxorubicin, the IC_50_ value ranged from 3.1 to 5.1 μg/mL (Mbaveng et al., 2011). The cytotoxicity of *B. abyssinica* ethanol leaves extract was studied using the MTT assay against the human diploid embryonic lung cells MRC-5, and the extract exhibited an IC_50_ value of 5.3 μg/mL (Zirihi et al., 2005). The *in vitro* cytotoxicity activities of *B. abyssinica* extracts in dichloromethane:methanol (1:1) and 5% aqueous methanol were studied using Vero type 199 kidney epithelial monkey cells and the MTT colometric assay technique. The extract exhibited CC_50_ values of 38.43 and 28.97 μg/mL, respectively (Omole et al., 2020). Hellebrigenin-3-acetate (**25**) and hellebrigenin-3,5-diacetate (**26**) showed significant cytotoxicity against KB cell culture at l0^−7^ and 10^–3^ μg/mL respectively. Hellebrigenin-3-acetate (**25**) showed significant inhibitory activity against intramuscular Walker carcinosarcoma 256 in rats at 8 mg/kg (Kupchan et al., 1968). The compounds (**21**, **23**, **25**, and **26**) are Cardiac glycosides in the category cardenolides have a plant-based origin (5-membered butyrolactone ring at 17th carbon), while bufadienolides produced from animals such as frog skin and carotid gland toads (6-membered pyrone-unsaturated lactone ring) are being used for anticancer activity. These glycosides all share a steroidal ring of 17 carbon atoms, an unsaturated lactone ring on the 17th carbon in beta conformation, and a sugar moiety on 3-OH. The sugar moiety, or glycine component of CGs, influences the drug’s pharmacokinetic properties, whereas the aglycone moiety causes pharmacological effects. They kill cancerous cells by inducing apoptosis, autophagy, and cell cycle arrest, and decrease the cytokine storm, which produces antiproliferative activity (Malik et al., 2022). The mechanism of action is presented in Figure 9.

#### 3.3.14 Excopula ejaculatory activity

In spinal male rats, the fictitious ejaculation of *B. engleriana* is examined. In the absence and presence of dopamine or oxytocin, anaesthetized rats were administered intravenously with aqueous and methanolic extracts from the dried leaves of *B. engleriana*. Dopaminergic and oxytocinergic pathways mediate the inhibitory impact of *B. engleriana* extracts on the expression of fictive ejaculation in spinal male rats. The possible use of *B. engleriana* in patients with fast ejaculation may be supported by this prolonged ejaculatory latency it causes (Watcho and Carro-Juarez, 2009).

#### 3.3.15 Acute toxicity

The acute toxicity of 80% methanol extracts of *B. abyssinica* leaves was evaluated on healthy Male Swiss albino mice. The crude extract caused no mortality in the first 24 h, as well as for the next 14 follow-up days, at a limit dose of 2000 mg/kg. This suggests that the extract’s median lethal dose (LD_50_) is greater than 2000 mg/kg (Kifle and Enyew, 2020).

## 4 Conclusion and future perspective

This review provides overall information on the Bersama genus, including traditional uses, chemical constituents, biological activity, and toxicity studies until February 2023. In terms of chemistry, 59 compounds’ structures were described and presented, with a focus on cardiac glycosides, terpenoids, steroids, and flavonoids. Meanwhile, we expounded the biological activity of isolated compounds as well as extracts, because the chemical compounds of the Bersama genus demonstrated a wide range of pharmacological actions. Therefore, throughout our review, we noticed that it has anti-tumor, antibacterial, anti-inflammatory, antiviral, antioxidant, anti-gonorrheal, antimalarial, antiproliferative, and other pharmacological properties. The genus provided remarkable drug-lead compounds such as paulliniogenin A (**9**), 16β-formyloxybersamagenin-1,3,5-orthoacetate (**11**), isoquercetrin (**39**), hyperoside (**35**), quercetin-3-*O*-arabinopyranoside (**40**), and mangiferin (**46**) with cytotoxicity and antioxidant activity.

Based on the present encouraged findings, the next stage of scientific activities will be the focus of attention. First and foremost, while there are eight species in the genus Bersama, only five (*B. swinnyi*, *B. stayneri*, *B. tysoniana*, *B. lucens*, and *B. abyssinica*) have been used as traditional folk medicines (Table 2), indicating that there are a few gaps in the traditional medicine of other species that need to be investigated further. Secondly, the acute toxicity of *B. abyssinica* was found to be safe with a lethal dose (LD_50_) greater than 2000 mg/kg. However, previous study has shown that the leaves of *B. abyssinica* sub-sp abyssinica are toxic to cattle and rabbits, and a crude extract is toxic to mice. This contradicts the plant’s traditional uses. The dosage is crucial for the ethnomedicinal uses of this plant, and it provides an outline for other Bersama genera, the toxicological evaluation must be thoroughly studied for their applications. Thirdly, only four species (*B. abyssinica*, *B. engleriana*, *B. lucens*, and *B. swinnyi*) were investigated and analyzed for their secondary metabolites (Table 3). Inadequate study on secondary metabolites in Bersama plants hinders their development and application. Plants from the Bersama genus should undergo in-depth phytochemical investigations along with bio-guided isolation. Fourthly, the majority of pharmacological records focused on crude extracts of Bersama plants, with no surface investigation of secondary metabolites. More study on the pharmacology, structure-activity relationship, and mechanisms of Bersama plants’ chemical constituents is needed to support their traditional use. Fifthly, cardiac glycosides and terpenoids are the major bio-constituents of the genus Bersama plants and further research is needed on the chemicals, biological effects and mechanisms. The toxicology of practically all extract and purified compounds has not been evaluated. This significantly restricts their potential as treatments in the future. Detailed investigations should be necessary. Lastly, plants of the genus Bersama are important folk medicines, containing several secondary metabolites and showing many pharmacological effects. Importantly, the pharmacological effects and mechanism of bio-constituents related to the traditional uses in folk medicines should be extensively discussed in subsequent studies.

In conclusion, 59 secondary metabolites have been isolated and identified from Bersama plants, some of which had antioxidant and antiproliferative activity, but these findings are insufficient to explain the conventional use of Bersama. Additionally, this review extensively discusses the relevance of the chemical constituents, modern pharmacological effects, and traditional uses in folk medicines. In the future, in-depth investigations on the bio constituents, mechanisms, toxicity, pharmacokinetics, and clinical trials to provide better scientific connotations of plants of the genus Bersama. The information in this review will guide the rational utilization and future development of Bersama species.
