# Supplementary material for: The ethnomedicine, phytochemistry, and pharmacological properties of the genus Bersama: current review and future perspectives
Source: Front Pharmacol. 2024 Mar 21;15:1366427. doi: 10.3389/fphar.2024.1366427 (PMC10991763; doi:10.3389/fphar.2024.1366427)
Supplement: Supplementary file 1 [file Table1.docx]

Table S1: Summary of pharmacological studies of *Bersama* genus

| Activity | Plant species | Plant part | Extract | Isolated Compound | Mode of action/Method | Effects | Ref |
| --- | --- | --- | --- | --- | --- | --- | --- |
| Antibacterial | *B. abyssinica* | Stem bark | MeOH | - | Disc diffusion assay | The methanol stems bark extracts demonstrated an inhibition zone of 16 mm against *K. pneumonia* and *P. aeruginosa*, which is comparable to the standard antibiotic ampicillin, which showed an inhibition zone of 21 to 23 mm. | (Ong'era et al. 2017) |
| Antibacterial | *B. abyssinica* | Stem bark | CH_2_Cl_2_ | Sitosterol 3-*O*-glucopyranose (**1**) | Disc diffusion assay | The compound exhibited moderate activity against *S. aureus* with inhibition zones of 14 and 13 mm for *K. pneumoniae*, respectively, and inhibition zones of 10 and 15 mm for *V. cholerae* and *E. coli* respectively | (Ong'era et al. 2017) |
| Antibacterial | *B. abyssinica* | Stem bark | CH_2_Cl_2_ | Lupeol (**7**) | Disc diffusion assay | The Compoud showed modest activity against *S. aureus*, with an inhibition zone of 14 mm and it also had inhibition zones of 11 and 8 mm against *B. subtilis* and *E. coli*, respectively | (Ong'era et al. 2017) |
| Antibacterial | *B. abyssinica* | Root | DCM/MeOH (1:1) and MeOH | - | Disc diffusion | The extract had an inhibition zone ranging from 11 to 13.6 mm against *E. coli*, *S. thyphimerium, S. aureus*, *and B. subtlis*, which was less active in both solvent systems than the standard drug ciprofloxacin, which had an inhibition zone ranging from 26 to 34 mm. | (Lemilemu et al. 2020). |
|  |  |  |  |  |  |  |  |
| Antibacterial | *B. abyssinica* | Root | DCM/MeOH (1:1) | 7-hydroxy-β-sitosterol (**3**) | Disc diffusion | The compound showed promising antibacterial activity against *E. coli* and *S.aureus*, with zones of inhibition of 12.6 and 12.5 mm, respectively | (Lemilemu et al. 2020) |

Table 4. *Continued*

| Antibacterial | *B. abyssinica* | Leaves | MeOH | - | Disc diffusion | The extract showed MIC of 25 mg/mL against *Xanthomonas campestris pv musacearum* bacteria which is less than that of the standard drug tetracycline, with MIC of 0.02 mg/mL | (Yemata et al., 2019) |
| --- | --- | --- | --- | --- | --- | --- | --- |
| Antibacterial | *B. abyssinica* | Roots | EtOH | - | Disc diffussion | The extract exhibited agaist with *S. Typhimurium*, *S. Typhi*, and *P. aeruginosa* with MIC values of 10, 10 and 2.5 mg/mL respectively, which is modest activity when compared to ciprofloxacin's MIC value of 0.025 mg/mL | (Bolou et al. 2011). |
| Antibacterial | *B. abyssinica* | Tender foliage | ethyl acetate | - | disc diffusion | The extract exhibited against *E.coli*, *K. pneumonia*, *S. aureus*, and *E. faecalis* with MIC values ranged from 12.5 to 100 mg/mL | (Ameya et al. 2019) |
| Antibacterial | *B. abyssinica* | leaves stem bark and root bark | Methanol | - | Disc diffusion | The extracts' MIC values varied from 0.19 to 0.78 mg/mL against *M. madagascariense* and *M. indicuspranii*, which is more comparable to the standard rifampicin's MIC value of 0.19 mg/mL | (Mwambela et al. 2014) |

Table 4. *Continued*

| Antibacterial | *B. lucens* | Barks | Water, ethyl acetate and ethanol | - | Micro plate | The water and ethanol extracts exhibited activities against tested pathogens *K. pneumoniae*, *B. subtilis*, *S. aureus* and *E. coli*  with minimum inhibitory concentration (MIC) values ranging from 3.1 mg/mL to >12.5 mg/mL | (Buwa and Van Staden 2006) |
| --- | --- | --- | --- | --- | --- | --- | --- |
| Antibacterial | *B. lucens* | Barks | CH_2_Cl_2_ and MeOH | - | Micro-titer plate | The extracts exhibited against *B. cereus*, *E. faecalis*, *E.coli*, *S. typhimurium* and *S. sonnei* with MIC values ranging from 0.3 mg/mL to 2.0 mg/mL in comparison to MIC values of 0.02 μg/mL to 0.07 μg/mL exhibited by the positive control | (Khumalo 2018) |
| Antiviral | *B. abyssinica* | Root bark | MeOH | - | MTT assay | The extracts inhibited HIV-1 replication at 50% effective concentrations (EC_50_) of 3.1 mg/mL with a corresponding selectivity index of 3.8 | (Asres et al. 2001) |
| Antiviral | *B. engleriana* | Leaves, barks, and roots | MeOH | - | Non-radioactive HIV-RT colorimetric ELISA kit | The extracts showed anti-reverse transcriptase activity IC_50_ values of 11.95, 18.75, and 9.38 μg/mL indicated moderate activity in comparison to the positive control drug doxorubicin, whose IC_50_ value was 4.24 μg/mL | (Mbaveng et al. 2011) |

Table 4. *Continued*

| Antiviral | *B. abyssinica* | Stem bark | Water | - | MTT | The extract showed IC50 values of 16 and 50 μg/mL against Delta B1 Covid-19, producing 75% virus mortality with negligible cytotoxicity effects on host cells | (Zekeya, Mamiro et al. 2022) |
| --- | --- | --- | --- | --- | --- | --- | --- |
| Antidiabetic | *B. engleriana* | Leaves | MeOH | - | Nicotinamide/STZ-induced diabetic adult male Wistar rats | The extracts suppressed blood glucose concentrations by 80.31% at a dose of 600 mg/kg, which was more effective than the standard drug glibenclamide (58.65%) | (Pierre et al. 2012) |
| Antidiabetic | *B. abyssinica* | Leaves | 80% Methanol | - | 3,5-dinitrosalicylic acid | The extract inhibited α-amylase enzyme with an IC_50_ of 6.57 μg/mL, which is comparable to acarbose's IC_50_ of 2.26 μg/mL | (Kifle and Enyew 2020) |
| Antidiabetic | *B. engleriana* | Leaves | MeOH | - | streptozotocin/nicotinamide (STZ-NA)-induced type 2 diabetic | The extract at 600 mg/kg showed to be the most effective; HDL-C levels were significantly higher after four weeks compared to untreated diabetic rats, and the effects were greater (p˂ 0.001) than glibenclamide (0.25 mg/kg) | (Pierre et al. 2012) |
| Antidiabetic | *B. abyssinica* | Leaves | Solvent fractions (aqueous and ethyl acetate) | - | normoglycemic mice | The solvent fraction showed percentage reduction in baseline blood glucose levels was 25.90%, 26.36%, 38.43%, 30.96%, and 49.42% for EAF200mg/kg, AQF200mg/kg, EAF400mg/kg, AQF400mg/kg, and GLC 5mg/kg, respectively | (Kifle et al. 2020) |
| Antifungal | *B. abyssinica* | Leaves | Aqueous and ethanol | - |  | The extracts exhibited MIC values of 98 and 195 μg/mL, respectively against *A. flavus*, which produces aflatoxin B1 | (Bene et al. 2017) |
| Antifungal | *B. abyssinica* | Leaves, stem, bark, and root | Petroleum ether, ethyl acetate and methanol. |  |  | The extracts have MIC values of 0.19, 0.78, and 0.78 mg/mL respectively against the coffee pathogenic fungus *Gibberella xylarioides* | (Mwambela and Kilambo 2011) |

Table 4. *Continued*

| Antifungal | *B. engleriana* | Roots, stem barks, leaves, and wood | Methanol | - | Agar diffusion | The extracts showed MIC values ranging from 9.76 to 39.06 μg/mL and MBC values ranging from 19.53 to 78.12 μg/mL against *C. albicans* and *C. gabrata* which were less potent than the positive control, which had MIC and MBC values of 2.44 and 4.88 μg/mL, respectively | (Kuete et al. 2008). |
| --- | --- | --- | --- | --- | --- | --- | --- |
| Antifungal | *B. lucens* | Barks | Water, ethyl acetate and ethanol | - | Micro plate | The extracts exhibited activities against *Candida albicans* with MIC values ranging from 0.78 mg/mL to 12.5 mg/mL | (Buwa and Van Staden 2006) |
| Antioxidant | *B. abyssinica* | Leaves | 80% methanol and aqueous fraction | - | DPPH | The IC_50_ values for the crude extract and fraction were 5.35 and 3.43 μg/mL, respectively, which are comparable to the standard ascorbic acid IC_50_ value of 2.65 μg/mL | (Kifle and Enyew 2020) |
| Antioxidant | *B. engleriana* | Root , stem bark, leaves, and wood | MeOH | - | DPPH | The DPPH• scavenging activity revealed that the extract from the leaves was the most active, with a 93.71% inhibition rate at 1000 μg/mL | (Kuete et al. 2008) |
| Antioxidant | *B. abyssinica* | Leaves | MeOH | - | DPPH | The extract has an IC_50_ value of 7.5 μg/mL, which is considered moderate activity when compared to the positive control quercetin, which had an IC_50_ value of 18.2 μM | (Asres et al. 2006) |
| Antioxidant | *B. abyssinica* | Leaves | MeOH | Isoquercetrin (**39**),  Hyperoside (**35**), Quercetin-3-*O*-arabinopyranoside (**40**), and Mangiferin (**46**) | DPPH | The compounds had IC_50_ values of 23.7, 22.6, 20.7, and 15.9 μM, which are promising when compared to quercetin | (Asres et al. 2006) |

Table 4. *Continued*

| Antitumor | *B. engleriana* | Root, stem bark, leaves, and wood | MeOH | - | DPPH | Extracts from the roots (69.32%) and leaves (65.42%) exhibited considerable tumor-reducing efficacy | (Kuete et al. 2008) |
| --- | --- | --- | --- | --- | --- | --- | --- |
| Antimalarial | *B. abyssinica* | Leaves | 80% methanol and fractions (aqueous, ethyl acetate, and chloroform) | - | *Plasmodium berghei* ANKA infected mice | The crude extract (49.51%, P < 0.001), aqueous (47.69%, P < 0.001), ethyl acetate (41.89%, P < 0.001), and chloroform (38.21%, P < 0.001) exhibited the highest chemosuppression at 400 mg/kg dose, which was less than the standard drug, chloroquine (25 mg/kg), showed 100% chemosuppression. | (Alehegn et al. 2020) |
| Antimalarial | *B. abyssinica* | Leaves | EtOH | - |  | The extract showed IC_50_ value of 23.9 μg/mL against *P. falciparum*, which is less powerful than the IC_50_ value of 0.1 μg/mL for the positive control | (Zirihi et al. 2005) |
| Antimalarial | *B. abyssinica* | Stem bark | DCM/MeOH (1:1) | - | Semi-automated micro-dilution | The extract showed IC_50_ of 12.85 and 8.48 μg/mL against *P. falciparum* D_6_ and W_2_ strains, respectively, which was less potent than the positive control chlroquine, which had IC_50_ values of 0.00124 and 0.00153 μg/mL against D_6_ and W_2_ strains, respectively | (Omole et al. 2020) |
| Antimalarial | *B. abyssinica* | Leaves | aqueous | - | Guinea pig ileum with isotonic contractions and different concentrations of a standard spasmogen and histamine | The extract was found to antagonise the spasmogenic effect of histamine in a nonreversible manner | (Makonnen and Hagos, 1993). |

Table 4. *Continued*

| Antigonorrheal | *B. engleriana* | Bark | MeOH | - | Agar dilution | The extract shown substantial activity against ATCC 49226, WHO A (βL−), *NGCS*1 (βL−), *NGCS*3 (βL−), NGCS5 (βL + ) and *NGCS*5 (βL + ) with MIC values of 16 μg/mL; gentamicin's MIC values ranged from 0.5 to 32 μg/mL. | (Mbaveng et al. 2011) |
| --- | --- | --- | --- | --- | --- | --- | --- |
| Wound healing | *B. abyssinica* | Leaves | 80% MeOH | - | 5% and 10% w/w ointment in excision, incision, and burn wound models | On the excision wound healing model, the extract produced 5% (99.5%) and 10% (100%) wound contraction on the 16^th^ day of treatment, as well as 5% (18.8) and 10% (28.2) percent reduction in epithelization, which is comparable to the positive control nitrofurazone, which produced 100% wound contraction and 27.4% reduction in epithelization | (Taddese et al. 2021) |
| Anthelmintic | *B. abyssinica* | Leaves | MeOH and EtOH | - | egg hatch assay | When compared to alebendazole (0.005 mg/mL), which indicated 99.33 and 99.66% action, the mean percentage suppression of *Haemonchus contortus* egg hatching after 48 h exposure at 2 mg/mL was 95.67% and 89% at the same concentration, respectively | (Asian 2018) |

Table 4. *Continued*

| Antidiarrheal | *B. abyssinica* | Leaves | MeOH |  | Castor oil-induced diarrhea mice | The crude extract (70.83%) significantly suppressed Castor oil-induced intestinal motility by 70.83%, which is comparable to the positive control loperamide (75.0%) at 400 mg/kg | (Ayalew et al. 2022) |
| --- | --- | --- | --- | --- | --- | --- | --- |
| Anti-inflammatory | *B. lucens* | Leaves | aqueous and ethanol | - | cyclooxygenase assay | The extracts exhibited suitable activities with percentage inhibition ranging from 71.0% to 80.0%, which were higher than 75.0% exhibited by the positive control | (McGaw et al. 1997) |
| Excopula Ejaculatory | *B. engleriana* | Leaves | aqueous and methanolic | - | In the absence and presence of dopamine or oxytocin, anaesthetized rats | The possible use of *B. engleriana* in patients with fast ejaculation may be supported by this prolonged ejaculatory latency it causes | (Watcho and Carro-Juarez 2009) |
| Cytotoxicity | *B. abyssinica* | Stem bark | EtOH | - | brine shrimp | The extract had an LC_50_ value of 7.8 μg /mL, which was higher than the standard drug's LC_50_ value of 16.3 μg /mL | (Moshi et al. 2010) |
| Cytotoxicity | *B. abyssinica* | Stem bark | MeOH | Paulliniogenin A (**21**) and 16β-formyloxybersamagenin-1,3,5-orthoacetate (**23**) | MTT | The compounds Paulliniogenin A (**21**) and 16β-formyloxybersamagenin-1,3,5-orthoacetate (**23**) demonstrated cytotoxicity against the KB3.1 cell line with IC_50_ values of 1.4 and 1.6 μM, respectively, which were less potent than the standard drug, which had an IC_50_ value of 0.000056 μM | (Nyamboki et al. 2021) |

Table 4. *Continued*

| Cytotoxicity | *B. abyssinica* | Stem bark | MeOH | - | brine shrimp larvae | The extract showed an LC_50_ value of 29.64 μg/mL, which was less than the standard drug's LC_50_ value of 16.4 μg/mL | (Mwambela et al. 2014) |
| --- | --- | --- | --- | --- | --- | --- | --- |
| Acute Toxicity | *B. abyssinica* | Leaves | MeOH | - | healthy Male Swiss albino mice | The extract median lethal dose (LD_50_) is greater than 2000 mg/kg | (Kifle and Enyew, 2020) |
| Cytotoxicity | *B. engleriana* | Leaves | MeOH | - | XTT assay | The extract showed IC_50_ values of 100, 15.7, 50.8, 8.6, and 20.3 μg/mL respectively agaist THP-1, DU145, HeLa, MCF-7, and HepG_2_, when compared to doxorubicin, the IC_50_ value ranged from 3.1 to 5.1 μg/mL the methanol extract showed etter activities on DU145 and MCF-7 | (Mbaveng et al. 2011) |
| Cytotoxicity | *B. abyssinica* | Leaves | EtOH | - | MTT | The extract exhibited an IC_50_ value of 5.3 μg/mL against human diploid embryonic lung cells (MRC-5) | (Zirihi et al. 2005) |
| Cytotoxicity | *B. abyssinica* | Leaves | DCM/MeOH (1:1) and MeOH | - | MTT | The extract exhibited CC_50_ values of 38.43 and 28.97 μg/mL, respectively against Vero type 199 kidney epithelial monkey cells | (Omole et al. 2020) |
| Cytotoxicity | *B. abyssinica* | Stem bark | EtOH | Hellebrigenin-3-acetate (**25**) and hellebrigenin-3,5-diacetate (**26**) | MTT | The compounds showed significant cytotoxicity against KB cell culture at l0^-7^ and 10^-3^ μg./mL., respectively | (Kupchan et al. 1968) |
